# Chemical Screening Identifies Danofloxacin as a Self‐Renewal Agonist of Embryonic Stem Cells through Alleviation of HDAC1‐Mediated Deacetylation of *Tert* and *Prdm10*

**DOI:** 10.1002/advs.202500624

**Published:** 2026-09-27

**Authors:** Yan Zhang, Junxiang Ji, Baoxing Dong, Zhenhua Zhu, Xucheng Lv, Peng Chen, Wenqiang Liu, Min Zhou, Shou‐Dong Ye

**Affiliations:** ^1^ Center for Stem Cell and Translational Medicine School of Life Sciences and Medical Engineering Anhui University Hefei China; ^2^ Shanghai Key Laboratory of Maternal Fetal Medicine Shanghai Institute of Maternal‐Fetal Medicine and Gynecologic Oncology Clinical and Translational Research Center Shanghai First Maternity and Infant Hospital Frontier Science Center For Stem Cell Research School of Life Sciences and Technology Tongji University Shanghai China; ^3^ Translational Medical Center for Stem Cell Therapy and Institute for Regenerative Medicine Shanghai East Hospital School of Life Sciences and Technology Tongji University Shanghai China; ^4^ Department of Clinical Laboratory Shanghai Children's Hospital School of Medicine Shanghai Jiao Tong University Shanghai China; ^5^ Department of Critical Care Medicine The First Affiliated Hospital of University of Science and Technology of China Division of Life Sciences and Medicine University of Science and Technology of China Hefei China

**Keywords:** danofloxacin, embryonic stem cells, pluripotency, self‐renewal

## Abstract

While significant advances have been made in maintaining stem cell pluripotency, the successful establishment of embryonic stem cells (ESCs) from various animal species in vitro remains challenging. This highlights the need for further optimization of culture conditions. Through compound library screening, we identified Danofloxacin, a small molecule, as a promoter of mouse ESC stemness. Danofloxacin enhances the formation of undifferentiated colonies and the generation of chimeric mice. Mechanistically, it suppresses *HDAC1* expression and activity, resulting in increased acetylation of histone H3 at lysines 9 (H3K9ac) and 27 (H3K27ac), which in turn upregulates the expression of *Tert* and *Prdm10*. Functional assays revealed that overexpressing *Tert* or *Prdm10* mimics Danofloxacin's effect on ESC self‐renewal, whereas knockdown of either gene disrupts this process. Moreover, the elevated levels of H3K9ac and H3K27ac activate the PI3K/AKT and HIF‐1α signaling pathways, both of which contribute to the transcriptional upregulation of *Tert* and *Prdm10*. These findings offer new insights into the pluripotency regulatory network and propose potential strategies for isolating ESCs from other species in vitro.

## Graphical abstract

This study identified Danofloxacin as a novel supporter of the maintenance of mouse embryonic stem cell (ESC) pluripotency. It functions partially by inhibiting HDAC1, which increases specific histone acetylation marks and activates key genes like *Tert* and *Prdm10*. This maintains the ESCs’ capacity for self‐renewal, genetic manipulation, and full differentiation, including the ability to contribute to chimeric embryos and germline transmission.

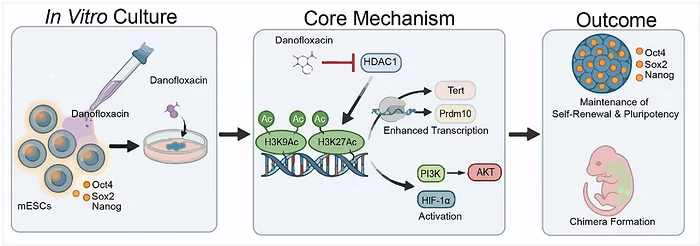

## 1 Introduction

The quest for optimal culture conditions for embryonic stem cells (ESCs) began in 1981 when Evans and Martin isolated mouse ESCs (mESCs) from 4.5‐day‐old embryos, demonstrating their diploid structure and differentiation potential [1, 2]. In 1984, Wobus made a significant breakthrough by introducing mitotically inactivated mouse embryonic fibroblasts (MEFs) for mESC culture, which enhanced stemness [3]. This method, which relies on inactivated MEFs and fetal bovine serum (FBS) [3], supports the long‐term self‐renewal of mESCs in vitro. A key cytokine secreted by MEFs is leukemia inhibitory factor (LIF) [4, 5], which maintains mESC stemness primarily through activation of the JAK/STAT3 signaling pathway. Several STAT3 targets, including *Tfcp2l1*, *Gbx2*, and *Klf4*, have been identified as crucial to this process [6, 7, 8]. However, culturing mESCs with serum and feeder cells limits our ability to conduct in‐depth analyses of the mechanisms underlying stem cell maintenance.

To overcome these limitations, researchers have increasingly turned to well‐defined small molecules to culture ESCs in vitro [9, 10, 11]. The use of these small molecules has enabled more efficient and cost‐effective research, advancing our understanding of stem cell biology. One notable discovery is the 2i combination—CHIR99021 (CHIR) and PD0325901 (PD). CHIR inhibits glycogen synthase kinase 3 beta (Gsk3β), while PD inhibits mitogen‐activated protein kinase kinase (MEK) [9]. CHIR activates the Wnt/β‐catenin pathway to reduce Tcf3 levels, a transcriptional repressor of pluripotency markers such as *Nanog* and *Esrrb* [12, 13, 14]. PD stabilizes the protein of Klf2 by inhibiting ERK, a direct substrate of MEK [15]. Other small molecules, such as GO6983 [16], CID755673 [10], and Spautin‐1 [11], have also shown promise in promoting ESC maintenance. While ESCs have been successfully derived from various species, including mice, rats, pigs, chickens, humans, and monkeys, only mouse and rat ESCs have been shown to generate germline‐competent chimeric animals when injected into embryos [17, 18, 19, 20, 21], a capacity that remains largely unexplored in other species. Therefore, developing new culture conditions to maintain ESC self‐renewal and pluripotency in vitro is a critical challenge.

In this study, using a *Rex1*‐GFP reporter mESC line, we identified Danofloxacin (DFA) from a small molecule library as a promoter of ESC pluripotency. DFA maintains green fluorescence in the *Rex1*‐GFP cell line and enhances the expression of pluripotency genes and the transcription of *Tert* and *Prdm10*. DFA treatment was accompanied by reduced HDAC1 expression and increased acetylation of histone H3 at lysines 9 (H3K9ac) and 27 (H3K27ac), suggesting a possible mechanistic link. This modification is enriched at the *Tert* and *Prdm10* loci, promoting their transcription and thereby supporting ESC self‐renewal.

## 2 Results

### 2.1 Compound Library Screening Identifies Danofloxacin as a Self‐Renewal‐Promoting Factor

**FIGURE 1:**
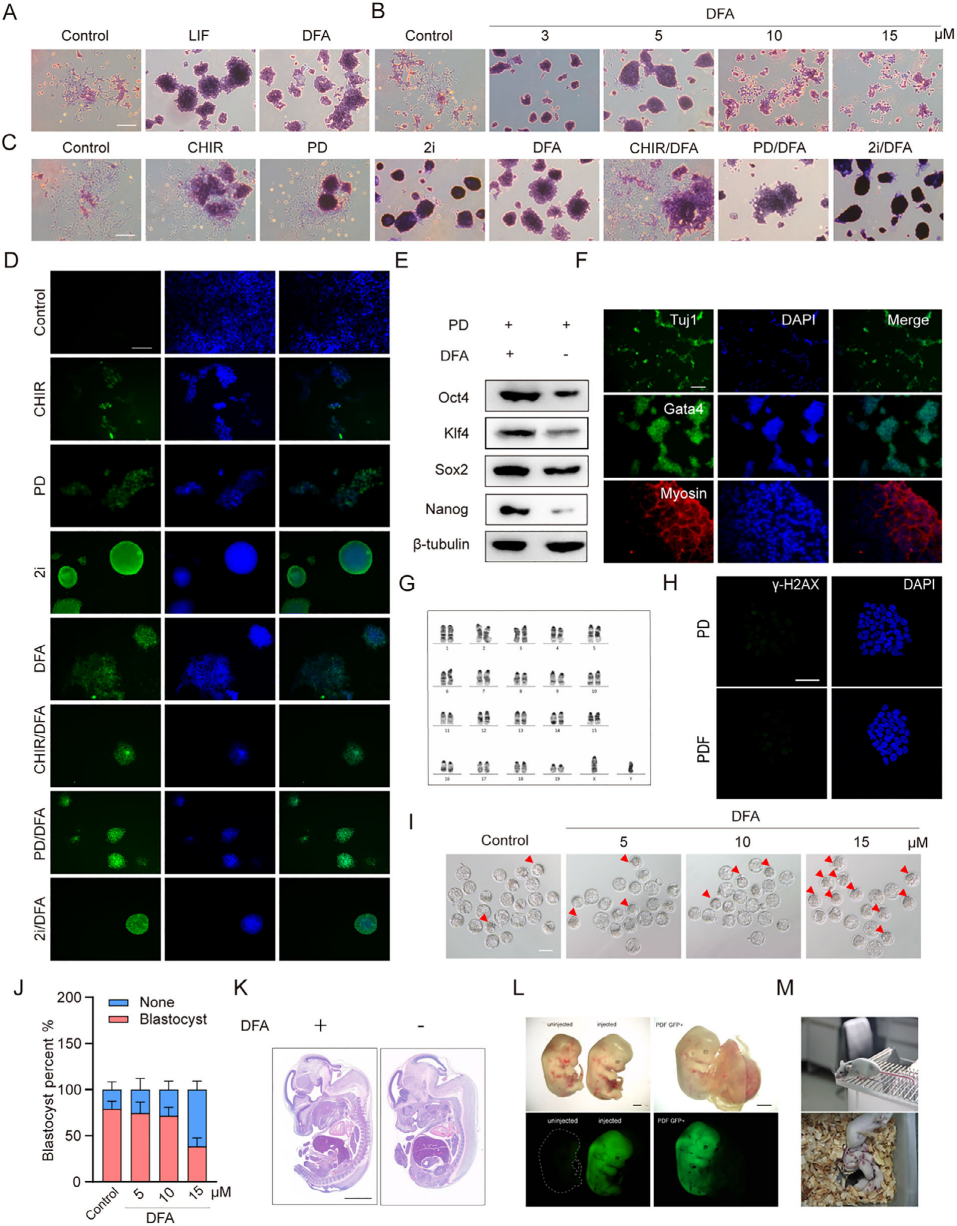
PD/DFA maintains self‐renewal and pluripotency in mESCs. (A) AP staining of mESCs cultured for 7 days in 10% serum‐containing medium supplemented with 5 µm DFA or 1000 U/mL LIF; medium without LIF served as the vehicle control. Scale bar: 100 µm. (B) AP staining of mESCs cultured for 7 days in 10% serum‐containing medium with a concentration gradient of DFA (3, 5, 10, and 15 µm); medium without LIF served as the vehicle control. Scale bar: 100 µm. (C) AP staining of mESCs treated for two passages with small‐molecule combinations (CHIR, PD, 2i, DFA, CHIR/DFA, PD/DFA, and 2i/DFA). Scale bar: 100 µm. (D) Immunofluorescence staining of the self‐renewal marker Oct4 in mESCs under the conditions described in (C). Scale bar: 100 µm. (E) Western blot analysis of pluripotency factors (Oct4, Klf4, Sox2, and Nanog) in mESCs cultured with PD or PD/DFA. β‐tubulin was used as the loading control. (F) Immunofluorescence staining of lineage‐specific markers (Tuj1, Gata4, and Myosin) in cells derived from embryoid bodies generated from PD/DFA‐treated mESCs. Scale bar: 100 µm. (G) Karyotype of mESCs maintained in PDF for 50 passages, showing a normal diploid chromosome number (2n = 40). (H) Immunofluorescence staining of γ‐H2AX in mESCs cultured under PD or PDF conditions to assess DNA double‐strand breaks and genomic stress. Scale bar: 50 µm. (I) Mouse blastocysts cultured in G1‐Plus medium with a concentration gradient of DFA (5, 10, and 15 µm); G1‐Plus medium alone served as the vehicle control. Red arrows indicate developmentally delayed blastocysts. Scale bar: 50 µm. (J) Quantification of developmental efficiency corresponding to (I). (K) H&E staining of E12.5 embryos derived from zygotes cultured to blastocysts in 5 µm DFA, with G1‐Plus medium used as the vehicle control. Scale bar: 2 mm. (L) E12.5 chimeric embryos generated from PDF‐treated mESCs. GFP^+^ mESCs were injected into E3.5 host blastocysts, which were transferred into pseudopregnant ICR mice. Embryos were collected at E12.5. GFP signals indicate chimeric contribution; non‐injected embryos served as negative controls. Scale bar: 2 mm. (M) Chimeric mice generated from injection of blastocysts with PDF‐treated mESCs, and progeny mice produced by the chimeras.

To identify novel factors that promote self‐renewal in mESCs, we screened a chemical library of 1,383 compounds targeting various cellular components. Each compound was added individually to the medium of *Rex1*‐GFP mESCs in the absence of LIF. After 7 days, GFP‐positive cells were observed in many of the DFA‐treated mESCs (Figure S1A). To validate these results, 46C mESCs were cultured for 7 days in basal mESC medium supplemented with either DFA or LIF, with cells cultured in basal medium without LIF serving as the control. Alkaline phosphatase (AP) staining, a marker of mESC identity, was then performed. LIF‐ and DFA‐treated mESCs exhibited enhanced AP activity, whereas the control group showed significant differentiation (Figure 1A; Figure S1B). These findings indicate that DFA promotes mESC self‐renewal. To determine the optimal concentration, four concentrations (3 µm, 5 µm, 10 µm, and 15 µm) were tested. After 7 days, mESCs treated with 5 µm DFA exhibited the strongest AP activity compared to other concentrations (Figure 1B; Figure S1C), making 5 µm the concentration selected for subsequent experiments. However, the undifferentiated state of mESCs diminished after three passages, suggesting that DFA cannot sustain stemness in vitro over prolonged periods.

To optimize DFA‐containing culture conditions, mESCs were cultured in basal medium supplemented with CHIR, PD, 2i, DFA, CHIR/DFA, PD/DFA, or 2i/DFA, with cells maintained in basal medium serving as the control. After two passages, we observed that the 2i‐containing conditions most effectively preserved the undifferentiated state of mESCs, as indicated by robust AP activity and sustained high expression of the self‐renewal marker Oct4 (Figure 1C,D; Figure S1D,E). Similar results were observed in mESCs treated with PD/DFA (PDF) or 2i/DFA (Figure 1C,D; Figure S1D,E). Western blot and RT‐qPCR analyses revealed increased expression of pluripotency genes, including *Klf4*, *Nanog*, *Oct4*, *Sox2*, *Gbx2*, *Tfcp2l1*, *Klf2*, and *Klf5*, under the PDF condition, compared to the PD‐only condition (Figure 1E; Figure S1F). To assess pluripotency under PDF conditions, cells were transferred to serum‐containing medium without PDF to form embryoid bodies (EBs). After 8 days, these cells were digested and plated on gelatin‐coated dishes for an additional 5 days. PDF‐treated mESCs retained their ability to differentiate into Tuj1+ neurons, Myosin+ cardiomyocytes, and Gata4+ primitive endoderm cells in vitro (Figure 1F). Cells cultured under PDF conditions could be stably maintained and passaged for 50 passages while retaining a normal karyotype (Figure 1G). Furthermore, immunostaining for phosphorylated histone H2AX revealed a reduced accumulation of DNA double‐strand breaks in both PD and PDF culture conditions (Figure 1H), indicating that PDF does not overtly increase genomic stress. Furthermore, an in vitro embryotoxicity assay was performed in which zygotes were cultured in G1‐Plus medium supplemented with DFA. At a concentration of 5 µm, DFA did not adversely affect blastocyst formation, with the efficiency of development to the blastocyst stage comparable to that of the control group cultured in G1‐Plus medium alone. In contrast, exposure to DFA at concentration of 15 µm resulted in varying degrees of developmental delay (Figure 1I,J). Blastocysts cultured under 5 µm DFA conditions were transferred into pseudopregnant ICR recipient females at 2.5 d.p.c. Embryos were harvested at E12.5 and subjected to hematoxylin and eosin (H&E) staining. Comparable to blastocysts cultured in G1‐Plus medium alone, blastocysts derived from DFA‐treated zygotes developed into morphologically normal E12.5 mouse embryos (Figure 1K). Furthermore, the PDF‐treated mESCs generated chimeric mice when injected into 3.5‐day post‐coitum blastocysts (Figure 1L,M; Figure S1G). These results demonstrate the potential of mESCs under PDF conditions for multi‐lineage differentiation.

To explore the effect of DFA on ESCs from other species, we tested human induced pluripotent stem cells (hiPSCs). Different concentrations of DFA were added to the culture medium of hiPSCs in the absence of Activin A and bFGF. After 5 days, hiPSCs treated with 2.5 µm DFA showed stronger AP activity and higher expression levels of SOX2, OCT4, and NANOG compared to other groups (Figure S2A–G). However, after three passages, all cells had fully differentiated, indicating that DFA only promotes short‐term self‐renewal in hiPSCs under these conditions.

### 2.2 Addition of Danofloxacin induces *Tert* and *Prdm10* transcription

**FIGURE 2:**
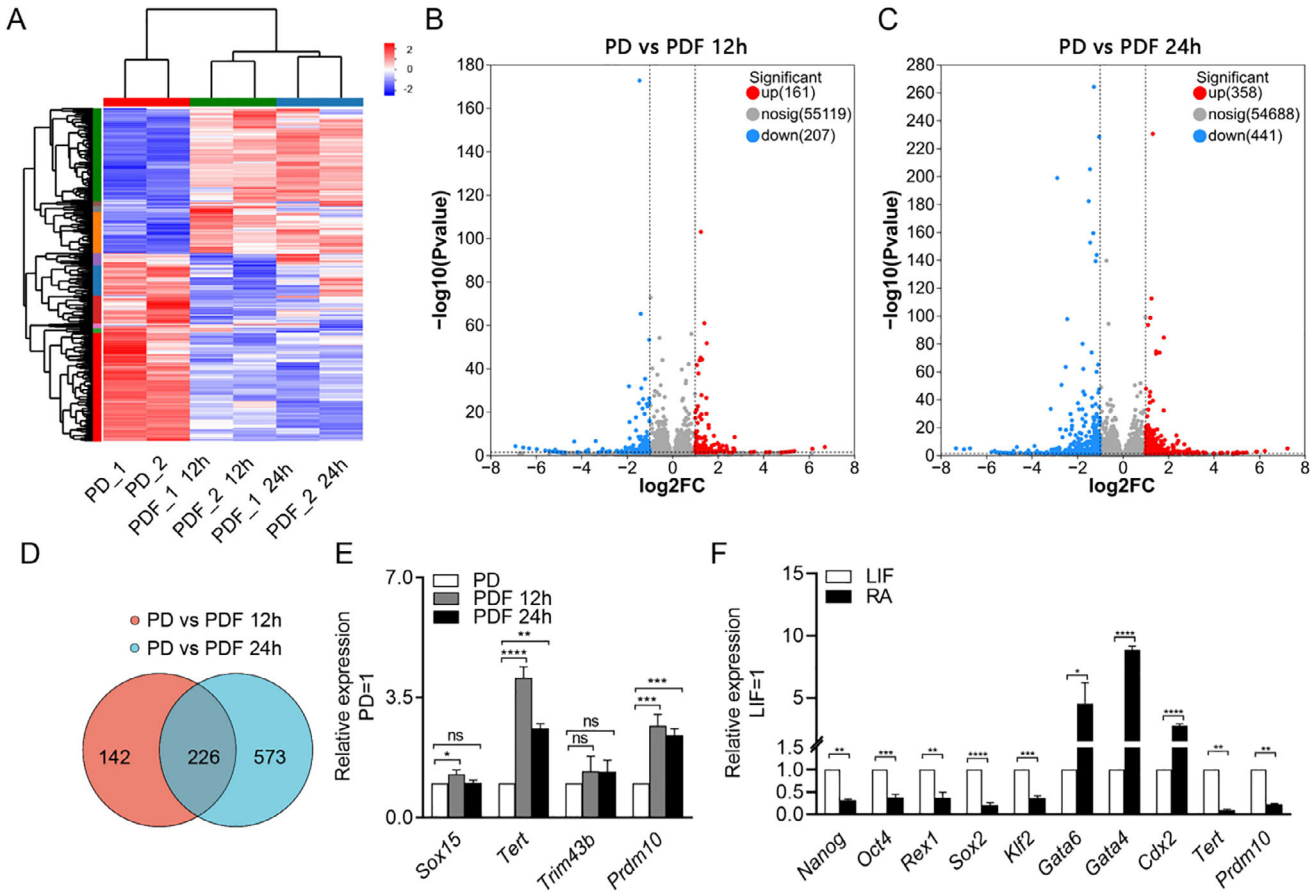
Transcriptomic profiling identifies Tert and Prdm10 as key targets of DFA. (A) Heatmap of gene expression profiles in mESCs treated with PD or PDF for 12 h and 24 h (two biological replicates per condition). (B, C) Volcano plots showing differentially expressed genes (DEGs) in mESCs cultured in PD versus PDF alone for 12 h (B) and 24 h (C). Significantly up‐ and down‐regulated genes are indicated by red and blue dots, respectively. (D) Venn diagram identifying 226 common DEGs in mESCs after 12 h and 24 h of PDF treatment compared to the PD control. (E) RT‐qPCR analysis of candidate genes (*Sox15*, *Tert*, *Trim43b* and *Prdm10*) in 46C mESCs after treatment with PDF for 12 h and 24 h (*n* = 3). (F) RT‐qPCR analysis of candidate genes (*Nanog*, *Oct4*, *Rex1*, *Sox2*, *Klf2*, *Gata6*, *Gata4*, *Cdx2*, *Tert*, *Prdm10*) in 46C mESCs after 4 days of RA treatment (*n* = 3). The data are presented as mean ± SD. Statistical analyses were performed using one‐way ANOVA (E) and Student's *t*‐test (F). ^*^ *p* < 0.05, ^**^ *p* < 0.01, ^***^ *p* < 0.001, ^****^ *p* < 0.0001.

To explore how DFA promotes self‐renewal, total RNA was extracted from mESCs treated with PD or PDF for various durations and subjected to high‐throughput sequencing. After 12 h of PDF treatment, we identified 161 upregulated and 207 downregulated genes (|log_2_FC| ≥ 1.000, p ≤ 0.05) relative to the PD control group. At 24 h, these numbers rose to 358 upregulated and 441 downregulated genes (Figure 2A–C). The Venn diagram illustrates the distribution of differentially expressed genes (DEGs) between the PD and PDF groups, revealing that 226 DEGs are shared between the two comparisons (Figure 2D). Among genes enriched in pathways related to mESC pluripotency, *Tert, Prdm10, Sox15, and Trim43b* were highlighted (Figure 2D,E). RT‐qPCR validation confirmed that *Tert* and *Prdm10* expression was significantly increased following PDF treatment at both 12 and 24 h (Figure 2E). Meanwhile, we also examined the expression of *TERT* and *PRDM10* in human iPSCs. The experimental results demonstrated that, following the addition of DFA, the expression levels of both *TERT* and *PRDM10* were significantly upregulated, consistent with the patterns observed in mice (Figure S2H). Based on these findings, we focused further investigation on *Tert* and *Prdm10*.

To assess the role of *Tert* and *Prdm10* in mESC self‐renewal, we cultured cells without LIF and treated them with retinoic acid (RA) to induce monolayer differentiation. After four days, RT‐qPCR analysis revealed a decrease in the expression of pluripotency‐associated genes (*Nanog*, *Oct4*, *Rex1*, *Sox2*, and *Klf2*), while differentiation‐associated genes (*Gata4*, *Gata6*, and *Cdx2*) were upregulated in the differentiated cells compared to LIF‐treated mESCs (Figure 2F). Notably, the expression of *Tert* and *Prdm10* was significantly reduced during differentiation (Figure 2F), suggesting their critical role in maintaining mESC stemness.

### 2.3 *Tert* and *Prdm10* Mediate the Effects of Danofloxacin on mESC Self‐Renewal

**FIGURE 3:**
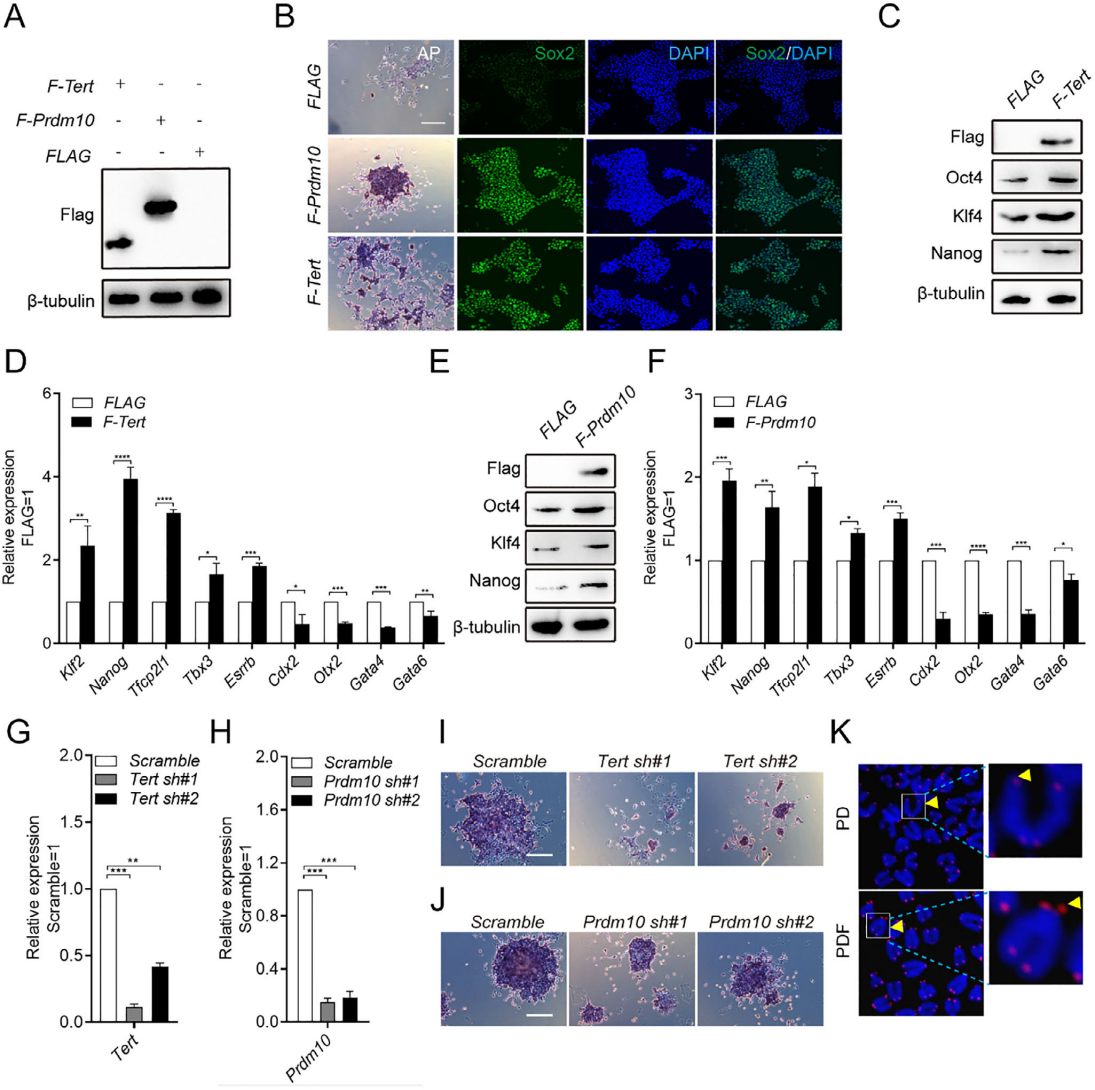
*Tert* and *Prdm10* mediate the effects of DFA on mESC self‐renewal. (A) Western blot analysis of Flag expression in 46C mESCs overexpressing *FLAG*, *F‐Tert*, or *F‐Prdm10*. β‐tubulin served as the loading control. (B) AP activity and immunofluorescence staining of Sox2 in *FLAG*, *F‐Tert*, and *F‐Prdm10* mESCs cultured in serum‐containing medium without LIF for 8 days. Scale bar: 100 µm. (C) Western blot analysis of Flag, Oct4, Klf4, and Nanog in 46C mESCs overexpressing *F‐Tert* or *FLAG* control. β‐tubulin was used as the loading control. (D) RT‐qPCR analysis of self‐renewal genes (*Klf2*, *Nanog*, *Tfcp2l1*, *Tbx3* and *Esrrb*) and differentiation genes (*Cdx2*, *Otx2*, *Gata4* and *Gata6*) in 46C mESCs overexpressing *F‐Tert* or *FLAG* (*n* = 3). (E) Western blot analysis of Flag, Oct4, Klf4, and Nanog in 46C mESCs overexpressing *F‐Prdm10* or *FLAG* control. β‐tubulin was used as the loading control. (F) RT‐qPCR analysis of self‐renewal genes (*Klf2*, *Nanog*, *Tfcp2l1*, *Tbx3* and *Esrrb*) and differentiation genes (*Cdx2*, *Otx2*, *Gata4* and *Gata6*) in 46C mESCs overexpressing *F‐Prdm10* or *FLAG* (*n* = 3). (G, H) RT‐qPCR analysis of *Tert* (G) and *Prdm10* (H) expression in 46C mESCs infected with *scramble* shRNA, *Tert* shRNA or *Prdm10* shRNA lentiviruses (*n* = 3). (I, J) AP staining of *scramble* shRNA, *Tert* shRNA or *Prdm10* shRNA mESCs cultured in DFA‐containing medium without LIF. Scale bar: 100 µm. (K) Representative telomere Q‐FISH images of metaphase chromosomes from mESCs treated with PDF or PD alone. Telomeres are shown in red; DAPI‐stained nuclei are in blue. The data are presented as mean ± SD. Statistical analyses were performed using Student's *t*‐test (D, F) and one‐way ANOVA (G, H). ^*^ *p* < 0.05, ^**^ *p* < 0.01, ^***^ *p* < 0.001, ^****^ *p* < 0.0001.

To investigate whether *Tert* and *Prdm10* can phenocopy the effects of DFA, we generated Flag‐tagged *Tert‐* or *Prdm10‐*overexpressing mESCs using the PiggyBac vector system (*F‐Tert* and *F‐Prdm10*), which effectively enhanced the expression of *Tert* and *Prdm10* (Figure 3A). An empty PiggyBac vector carrying Flag was used as a control (FLAG) (Figure 3A). These cell lines were seeded at low density in serum‐containing medium without LIF. After eight days, the *Tert‐* and *Prdm10‐*overexpressing cells exhibited increased AP activity and higher levels of pluripotency markers (*Sox2, Oct4, Klf4, Nanog, Klf2, Tfcp2l1, Tbx3, and Esrrb*) compared to FLAG control cells, with lower expression of differentiation‐associated genes (*Cdx2, Otx2, Gata4 and Gata6*) (Figures 3B‐F; Figure S3A,B). Furthermore, after culturing in PD‐containing medium for two passages, the *Tert‐* and *Prdm10‐*overexpressing cells formed more AP‐positive colonies than FLAG control cells (Figure S3C,D). These results indicate that enforced expression of *Tert* and *Prdm10* can promote the maintenance of the undifferentiated state of mESCs.

To examine whether knockdown of *Tert* or *Prdm10* impairs the function of DFA in mESCs, we designed two short hairpin RNAs (shRNAs) targeting *Tert* and *Prdm10* transcripts (*Tert sh#1, Tert sh#2, Prdm10 sh#1 and Prdm10 sh#2*). A construct with a nonspecific shRNA sequence (Scrambled shRNA) was used as a control. After infecting 46C mESCs with shRNA lentiviral particles for one week, we confirmed the successful downregulation of *Tert* and *Prdm10* by RT‐qPCR, and the cells were maintained in LIF/serum medium (Figure 3G,H). After two passages in DFA‐containing medium without LIF, *Tert* shRNA‐infected cells exhibited significantly reduced AP activity (Figure 3I; Figure S3E), while *Prdm10* shRNA cells showed partial differentiation (Figure 3J; Figure S3E).

Additionally, since Tert is a telomere‐associated gene influencing telomere lengthening [22, 23], we performed telomere Q‐FISH staining on cells cultured under PDF conditions and found that, compared with cells cultured only under PD conditions, cells under PDF conditions exhibited longer telomere lengths (Figure 3K). This is consistent with our observation of higher Tert expression under PDF conditions. Similarly, RT‐qPCR analysis revealed significantly longer telomeres (*Tel*) in *Tert*‐overexpressing cells compared to FLAG control cells (Figure S3F). Similar results were observed under DFA‐containing conditions (Figure S3G). Collectively, these findings suggest that the effect of DFA on mESC maintenance depends on *Tert* and *Prdm10*, with a close association to telomere maintenance.

### 2.4 Danofloxacin Inhibits the Expression and Activity of Histone deacetylase 1

**FIGURE 4:**
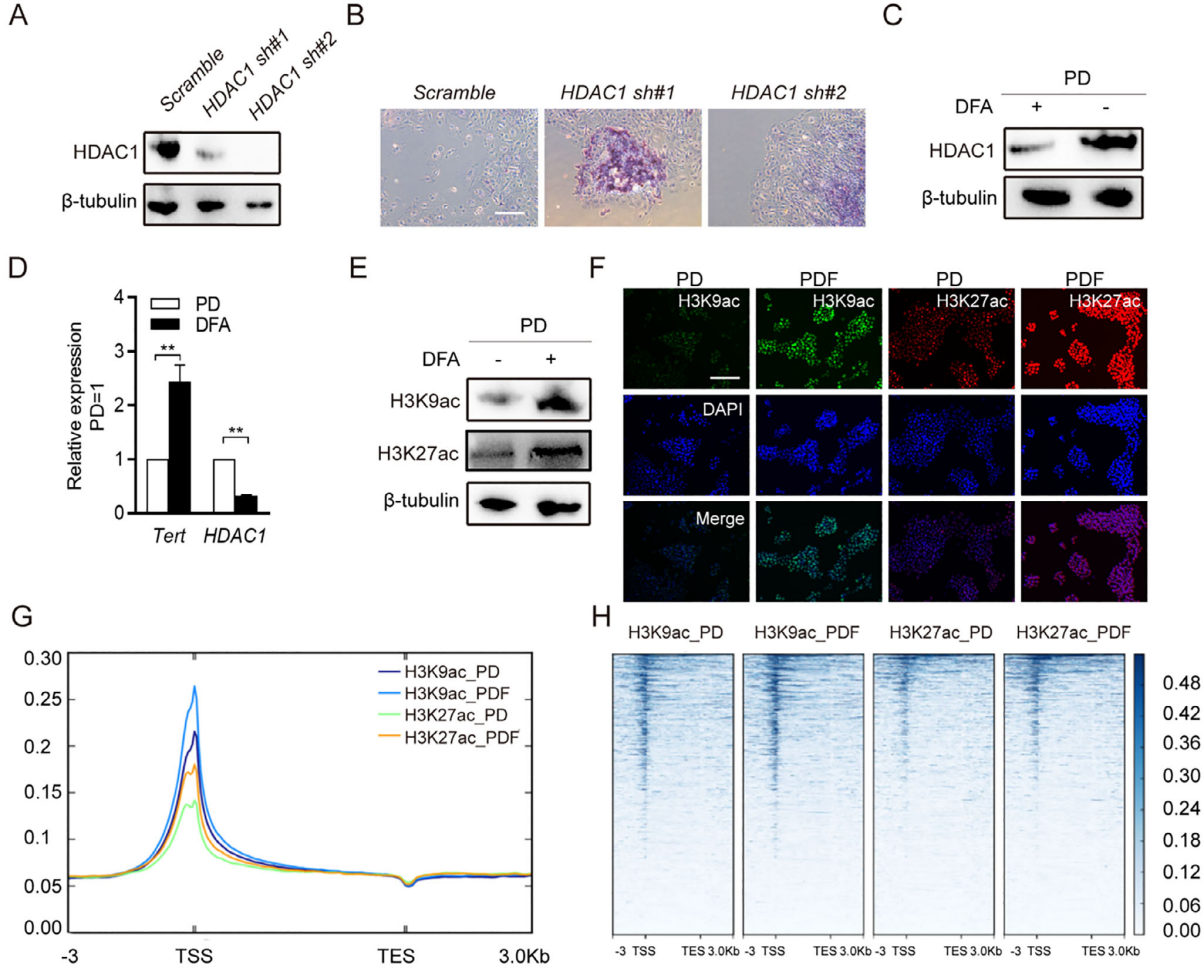
DFA suppresses HDAC1 and increases histone acetylation. (A) Western blot analysis of HDAC1 protein levels in scramble control and *HDAC1* shRNA mESCs. β‐tubulin served as the loading control. (B) AP staining of *scramble*, *HDAC1* sh#1, and *HDAC1* sh#2 mESCs cultured for 7 days in 10% serum‐containing medium without LIF. Scale bar: 100 µm. (C) Western blot analysis of HDAC1 protein levels in 46C mESCs treated with PD or PDF. β‐tubulin was used as the loading control. (D) RT‐qPCR analysis of *HDAC1* and *Tert* expression in 46C mESCs treated with PD or PDF for 12 h (*n* = 3). (E) Western blot analysis of H3K9ac and H3K27ac levels in 46C mESCs treated with PD or PDF. β‐tubulin was used as the loading control. (F) Immunofluorescence staining of H3K9ac and H3K27ac in 46C mESCs treated with PD or PDF. Scale bar: 100 µm. (G, H) Average signal intensity (G) and heatmaps (H) of H3K9ac and H3K27ac enrichment at transcription start site (TSS) regions, as determined by CUT&Tag sequencing (normalized by RPKM). The data are presented as mean ± SD. Statistical analyses were performed using Student's *t*‐test (D). ^**^ *p* < 0.01.

To investigate how DFA induces *Tert* and *Prdm10* expression, we first predicted its molecular structure using SMILES notation (Figure S4A). The structure was input into the Swiss Target Prediction database, which identified 100 potential target proteins, 27% of which were enzymes (Figure S4B). Among these, histone deacetylase 1 (HDAC1) stood out. mESCs with stable *HDAC1* knockdown exhibited partial self‐renewal after LIF withdrawal (Figure 4A,B; Figure S4C,D), suggesting HDAC1 could be a potential target of DFA. Next, molecular docking analysis was performed to assess the binding of HDAC1 and DFA, revealing multiple binding sites (Figure S4E,F). However, in vitro enzyme activity assays showed that, unlike trichostatin A (TSA), an HDAC inhibitor, DFA did not directly inhibit HDAC1 protein activity (Figure S4G). Despite this, the DFA‐treated group exhibited lower HDAC1 protein levels than the control group (Figure 4C; Figure S4H). RT‐qPCR analysis further confirmed that DFA suppressed *HDAC1* transcription (Figure 4D). Additionally, increased H3K9ac and H3K27ac levels were detected following DFA treatment, resembling the pattern observed with TSA treatment (Figure 4E,F; Figure S4I). CUT&Tag experiments using specific antibodies for H3K9ac and H3K27ac demonstrated enhanced DNA fragment binding after DFA treatment (Figure 4G,H). To contextualize these findings, we employed TSA as a functional reference to observe the kinetic accumulation of these acetylated marks. Similar to the robust increase induced by the reference inhibitor over a time course of 8 to 48 h, DFA administration effectively mimicked this hyperacetylation effect (Figure S4J,K). This suggests that while DFA does not directly bind the HDAC1 catalytic site, it effectively establishes a repressive chromatin state at the HDAC1 gene itself, ultimately leading to the sustained global elevation of H3K9ac and H3K27ac levels required for mESC self‐renewal (Figure S4J,K). To further investigate the epigenetic regulatory mechanism of DFA, we performed Chromatin Immunoprecipitation sequencing (ChIP‐seq) to profile various histone modifications across the HDAC1 gene locus. Our results revealed that DFA treatment (PDF group) led to a marked increase in the enrichment of H3K9me3 and H3K27me3, two hallmark repressive histone marks specifically at the HDAC1 promoter region. In contrast, the enrichment levels of H3K4me3, a modification typically associated with active transcription, showed no significant changes upon DFA administration (Figure S4L). These findings suggest that DFA specifically modulates inhibitory histone methylation to establish a more compact, transcriptionally repressive chromatin state at the HDAC1 promoter. We performed a dual‐luciferase reporter assay (Figure S4M). Our results demonstrated that DFA treatment (PDF group) significantly inhibited the luciferase activity driven by the *HDAC1* promoter compared to the control group (Figure S4N). Together, these results suggest that DFA exerts part of its effects in association with changes in HDAC1 activity and levels of H3K9ac and H3K27ac.

### 2.5 Induction of Histone Acetylation by Danofloxacin Enables Transcription of *Tert* and *Prdm10*

**FIGURE 5:**
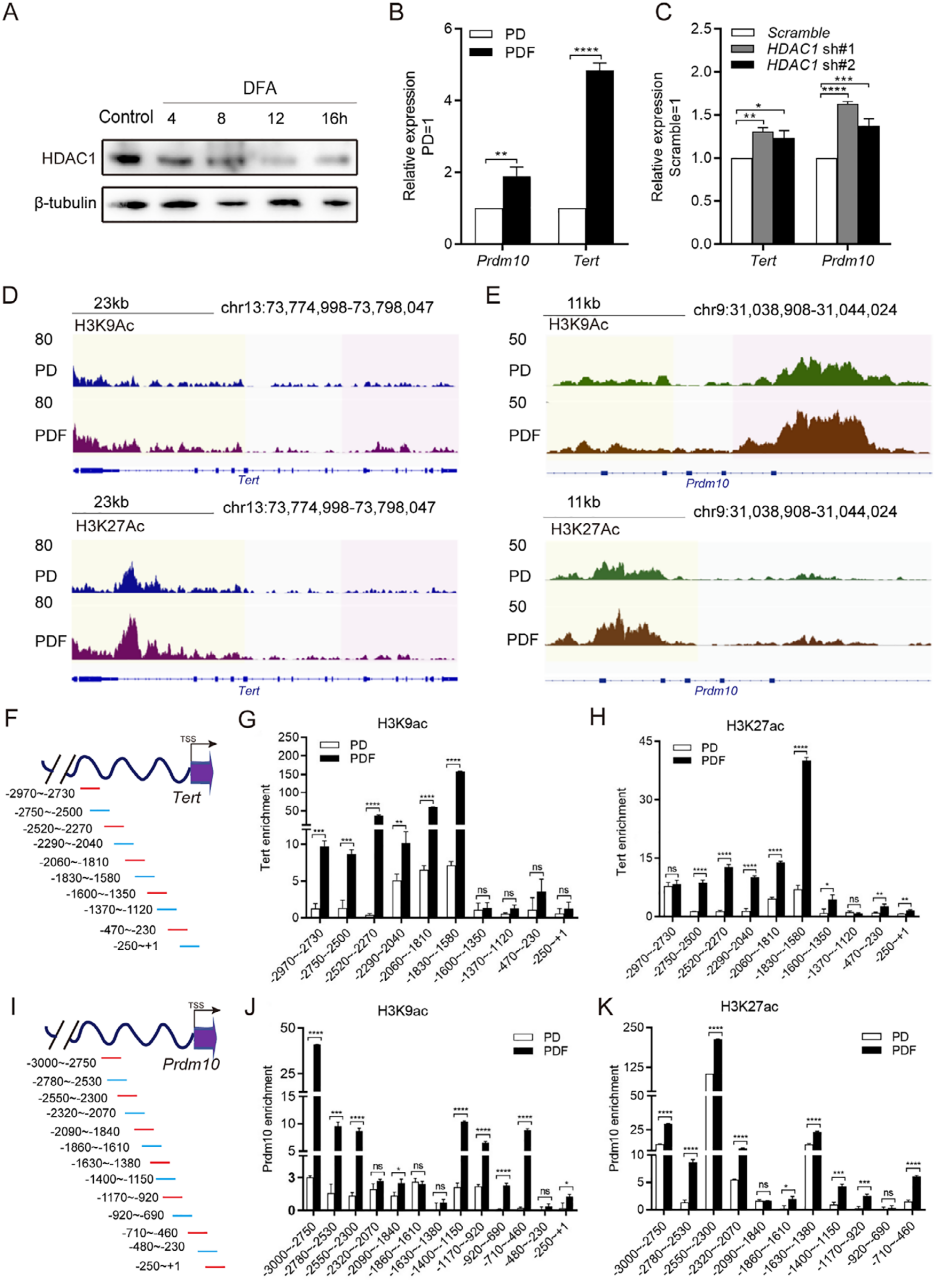
HDAC1 regulates the transcription of Tert and Prdm10. (A) Western blot analysis of HDAC1 protein levels in mESCs cultured in PD‐containing conditions in the presence or absence of DFA for 4 h, 8 h, 12 h, and 16 h. β‐tubulin served as the loading control. (B) RT‐qPCR analysis of *Tert* and *Prdm10* expression in 46C mESCs treated with PD or PDF for 12 h (*n* = 3). (C) RT‐qPCR analysis of *Tert and Prdm10* expression in *scramble* control and *HDAC1* shRNA 46C mESCs (*n* = 3). (D, E) IGV browser tracks showing H3K9ac and H3K27ac enrichment at the *Tert* (D) and *Prdm10* (E) genomic loci in mESCs treated with PD or PDF. (F) Schematic diagram of primer design spanning the *Tert* promoter region (approximately −3.0 kb to +1). (G, H) RT‐qPCR analysis of H3K9ac (G) and H3K27ac (H) enrichment at the specified region of the *Tert* promoter (*n* = 3). (I) Schematic diagram of primer design spanning the *Prdm10* promoter region (approximately −3.0 kb to +1). (J, K) RT‐qPCR analysis of H3K9ac (J) and H3K27ac (K) enrichment at the specified region of the *Prdm10* promoter (*n* = 3). The data are presented as mean ± SD. Statistical analyses were performed using Student's *t*‐test (B, G, H, J, K) and one‐way ANOVA (C). ^*^ *p* < 0.05, ^**^ *p* < 0.01, ^***^ *p* < 0.001, ^****^ *p* < 0.0001.

HDAC1 protein expression declined in a time‐dependent manner (Figure 5A; Figure S5A). Notably, at the 12‐hour time point, increased expression of *Tert* and *Prdm10* was observed in the presence of PDF (Figure 5B), pointing to a possible relationship between these changes. To validate this relationship, we performed HDAC1 knockdown experiments in mESCs using a lentiviral system. Knockdown of *HDAC1* (*HDAC1* sh#1 and *HDAC1* sh#2) resulted in increased expression of *Tert* and *Prdm10* compared to scrambled shRNA control cells (Figure 5C; Figure S5B), further supporting a possible association between HDAC1 levels and the expression of *Tert* and *Prdm10*.

Next, we analyzed the distribution of H3K9ac and H3K27ac modifications at the transcription start sites (TSS) of *Tert* and *Prdm10*. Following DFA treatment, there was an increase in the presence of H3K9ac and H3K27ac on the gpromoters of both genes (Figure 5D,E). To further explore this, we conducted ChIP experiments with antibodies against H3K9ac and H3K27ac in mESCs. RT‐qPCR analysis revealed that DFA treatment significantly increases H3K9ac and H3K27ac enrichment at the Tert and Prdm10 gene loci (Figure 5F–K). Together, these findings suggest that the effect of DFA is linked to reduced HDAC1 activity, accompanied by increased histone acetylation and transcriptional upregulation of *Tert* and *Prdm10*.

### 2.6 PI3K/AKT and HIF‐1α Signaling Pathways Contribute to Histone Acetylation‐Mediated Transcription of *Tert* and *Prdm10*

**FIGURE 6:**
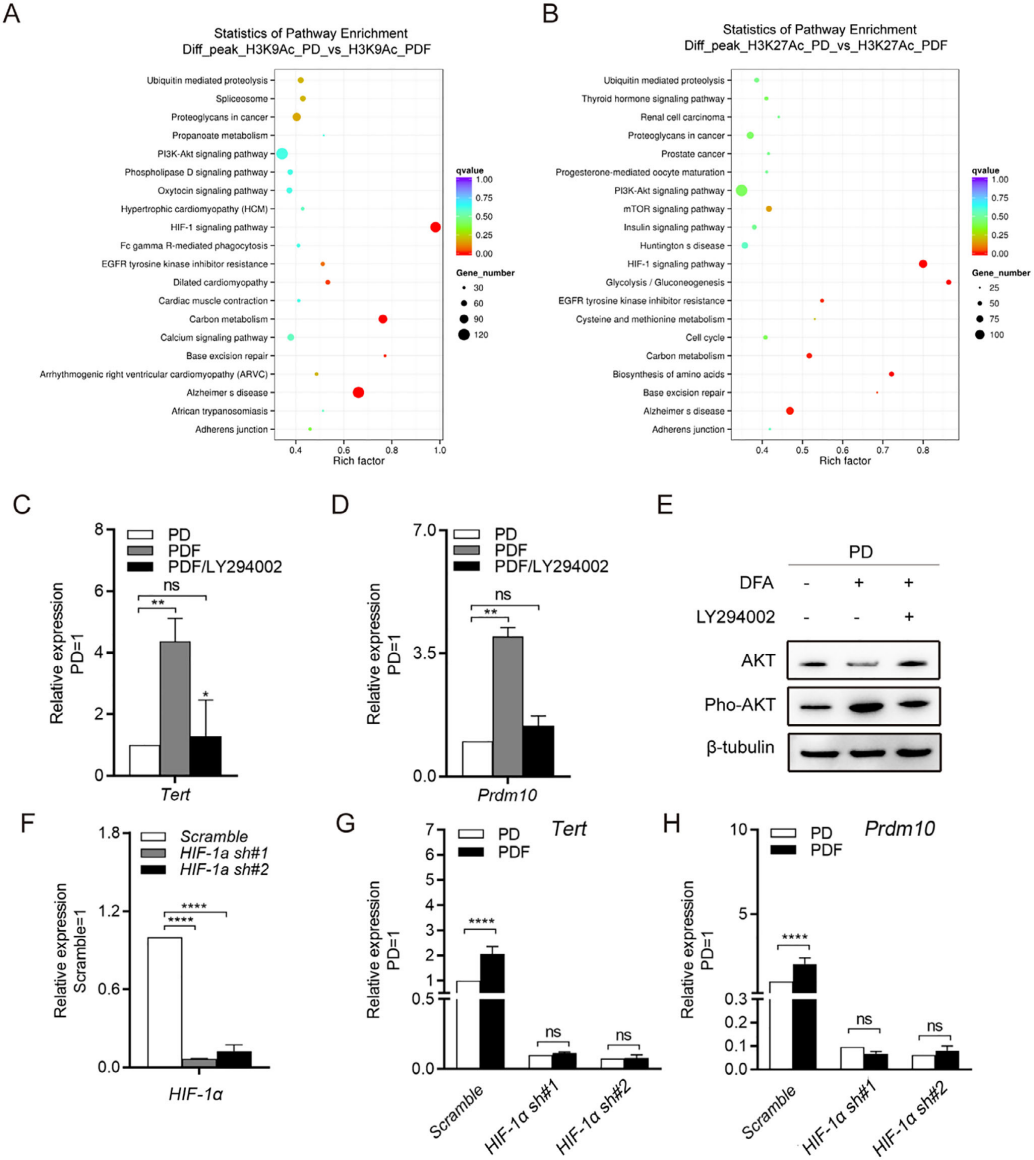
DFA activates PI3K/AKT and HIF‐1α signaling to promote the transcription of *Tert* and *Prdm10*. (A, B) KEGG enrichment analysis of the top 20 signaling pathways associated with H3K9ac (A) and H3K27ac (B) in mESCs treated with PD or PDF. (C, D) RT‐qPCR analysis of *Tert* (C) and *Prdm10* (D) expression in 46C mESCs treated with PD or PDF in the presence or absence of 10 µm LY294002 (*n* = 3). (E) Western blot analysis of total AKT and phosphorylated AKT (Pho‐AKT) levels in mESCs treated with PD or PDF in the presence or absence of LY294002. β‐tubulin served as the loading control. (F) RT‐qPCR analysis of *HIF‐1ɑ* expression in 46C mESCs infected with *scramble* shRNA or *HIF‐1ɑ* shRNA lentiviruses (*n* = 3). (G, H) RT‐qPCR analysis of *Tert* (G) and *Prdm10* (H) expression in *scramble* control and *HIF‐1ɑ* shRNA 46C mESCs cultured in PD or PDF (*n* = 3). The data are presented as mean ± SD. Statistical analyses were performed using one‐way ANOVA (C, D, F) and two‐way ANOVA (G, H). ^**^ *p* < 0.01, ^****^ *p* < 0.0001.

KEGG analysis of CUT&Tag sequencing data for H3K9ac and H3K27ac enrichment in mESCs cultured under DFA‐containing conditions revealed significant activation of the PI3K/AKT and hypoxia‐inducible factor‐1α (HIF‐1α) signaling pathways (Figure 6A,B). To assess the involvement of these pathways in the expression of *Tert* and *Prdm10*, we treated cells with LY294002, a specific inhibitor of PI3K, and observed a marked reduction in the expression of both genes following AKT pathway inhibition (Figure 6C,D). In agreement with these results, both DFA and TSA treatment increased AKT phosphorylation (Figure 6E; Figure S6A,B), suggesting that the enhancing effect of DFA on *Tert* and *Prdm10* expression may, at least in part, be associated with activation of the PI3K/AKT signaling pathway. Furthermore, to evaluate the role of HIF‐1α, we downregulated its expression using shRNA lentiviruses (Figure 6F). This was accompanied by a significant reduction in *Tert* and *Prdm10* expression (Figure 6G,H), suggesting that HIF‐1α may also be involved in the gene activation observed following DFA treatment. In conclusion, our data indicate that DFA‐induced changes in H3K9ac and H3K27ac were accompanied by alterations in the PI3K/AKT and HIF‐1α signaling pathways, along with increased expression of *Tert* and *Prdm10*.

## 3 Discussion

In this study, we established a novel culture condition by introducing a small molecule DFA to promote the maintenance of mouse and human ESC pluripotency. Although DFA is primarily recognized as a fluoroquinolone antibiotic [24], its mechanism of action in ESCs remains poorly understood. To address this, we employed high‐throughput sequencing and molecular cell biology experiments, which revealed that DFA treatment was associated with the upregulation of two key genes involved in mESC fate decision, *Tert* and *Prdm10* (Figure 3A‐J). Telomerase, consisting of the catalytic subunit *Tert* and the RNA component *Terc*, is crucial for telomere repair and elongation [25, 26, 27]. *Terc* is widely expressed, while *Tert* expression is tightly regulated. In ESCs, *Tert* is highly expressed, whereas in somatic cells, both *Tert* expression and telomerase activity are often low or undetectable [28]. Telomeres are specialized structures at the ends of chromosomes, and the absence of functional telomeres significantly impairs pluripotency in iPSCs, preventing teratoma formation [29]. mESCs without *Tert* exhibit abnormal differentiation [30], due to the global changes in chromatin accessibility and gene expression [31]. Similarly, *Tert* knockout in human ESCs reduces their proliferative capacity [32]. Notably, *Tert* expression in human ESCs is synergistically activated by transcription factors *Sp1* and *c‐Myc*, which are closely linked to stem cell fate commitment [33]. These findings underscore the importance of *Tert* in regulating ESC fate decisions and maintaining pluripotency. In our study, we found that DFA treatment was accompanied by upregulation of *Tert*, a change that contributes to the maintenance of mESC stemness (Figure 3A–D), with *Tert* expression gradually decreasing as cells differentiate (Figure 2F). Unlike *Tert*, the molecular and functional properties of *Prdm10*—also known as tristanin and a member of the same phylogenetic subfamily as *Prdm15*—are not as well characterized [34]. *Prdm10* contains an N‐terminal PR domain, ten C2H2 zinc fingers, and a C‐terminal glutamine‐rich transactivation domain. It is expressed in various embryonic and adult tissues [35]. Homozygous deletion of *Prdm10* in mice results in embryonic lethality [36], with developmental arrest occurring before blastocyst formation [37]. Similarly, acute deletion of *Prdm10* in mESCs leads to reduced growth and increased apoptosis [37]. Notably, the effects of *Prdm10* deficiency are partly mediated through *EIF3B*‐dependent global translation regulation [37]. Moreover, *Prdm10* influences *Bcl‐2* expression: depletion of *Prdm10* reduces *Bcl‐2* levels, while overexpression of *Prdm10* promotes *Bcl‐2* expression [38]. Luciferase reporter assays have shown that *Prdm10* binds to the *Bcl‐2* promoter in vivo [38]. In our study, we observed that *Prdm10* is activated alongside Tert upon DFA treatment (Figure 3A–K), highlighting its critical role in maintaining the undifferentiated state of mESCs.

HDAC1 appears to play a role in DFA‐mediated transcription of *Tert* and *Prdm10*. DFA suppresses the expression of HDAC1 (Figure 4C,D), and this regulatory effect is associated with the increased H3K9me3 and H3K27me3 levels at the HDAC1 promoter (Figure S4L). HDAC1 is a member of class I histone deacetylases, which also include HDAC2, HDAC3, and HDAC8 [39]. These enzymes negatively regulate histone acetylation by removing acetyl groups from histone tails [40]. In mESCs, HDAC1 binds to the regulatory regions of numerous genes, many of which are upregulated upon *HDAC1* depletion, including key regulators of ESC self‐renewal and differentiation [41]. Notably, the effects of complete *HDAC1* knockout differ from partial inhibition of its activity in mESCs. HDAC1 is essential for early embryonic development, as loss of it leads to early embryonic lethality, but ESCs can maintain pluripotency in the absence of *HDAC1* or its family member *HDAC2* [42], although the expression of pluripotency genes such as *Oct4*, *Nanog*, *Sox2*, and *Rex1* is reduced in these cells [43, 44]. Additionally, ESCs deficient in *HDAC1* exhibit smaller embryoid bodies with abnormal differentiation patterns, including spontaneous rhythmic contraction and increased expression of cardiomyocyte and neuronal markers [42]. Interestingly, inhibition of HDAC activity using specific inhibitors such as TSA, valproic acid (VPA), or butyrate promotes ESC self‐renewal in various species and enhances the reprogramming efficiency of iPSCs [45, 46, 47, 48, 49, 50]. Consistent with these findings, our study showed that DFA treatment was accompanied by increased H3K9ac and H3K27ac levels, along with elevated transcription of *Tert* and *Prdm10* (Figure 5F–K). Notably, HDAC1 suppression represents one of the primary mechanisms through which DFA exerts its effects, but it is not the sole biochemical property of this compound. Genome‐wide sequencing using CUT&Tag revealed that DFA‐induced histone acetylation can stimulate the PI3K/AKT and HIF‐1α signaling pathways (Figure 6A,B). This is in line with previous reports demonstrating that the PI3K/AKT pathway can upregulate *Tert* expression in mice [51]. DFA may activate the PI3K/AKT pathway by regulating upstream regulatory genes of this pathway, and this is further corroborated by the observation that HDAC1 silencing promotes AKT phosphorylation in mouse myotubes, human mesenchymal stem cells, and acute myocardial ischemia and reperfusion injury [52, 53, 54]. Additionally, activation of the PI3K/AKT signaling pathway has been shown to maintain self‐renewal and support multipotent differentiation in both human and mouse pluripotent stem cells [55, 56]. Moreover, HIF‐1α, a master transcription factor regulating gene expression in stem cell metabolism and behavior, has been implicated in *Tert* regulation [57, 58]. Overexpression of exogenous *HIF‐1α* cDNA in murine ESCs promotes cardiogenesis [59]. HIF‐1α also plays a critical role in the metabolic and functional transition of ESCs to the epiblast stem cell‐like stage [60]. In addition, upregulation of *HIF‐1α* inhibits mESC self‐renewal by negatively regulating the LIF/Stat3 pathway through direct binding to the reverse hypoxia‐responsive element located in the LIFR promoter [60]. In contrast, suppression of *HIF‐1α* facilitates the maintenance of ESCs in their undifferentiated state under hypoxia in vitro [61]. We found that DFA treatment was accompanied by increased H3K9ac and H3K27ac levels in mESCs, along with changes in the PI3K/AKT and HIF‐1α pathways and increased expression of *Tert* and *Prdm10*.

Notably, several key aspects remain unresolved in this study. First, the upstream molecular events by which DFA represses HDAC1 transcription remain undefined; complementary approaches, including CRISPR‐based screening and chromatin conformation assays, will be valuable for identifying the direct effectors involved. Second, whether increased histone acetylation directly activates the PI3K/AKT and HIF‐1α pathways or represents a parallel downstream event remains unclear. Additionally, the mechanisms by which AKT and HIF‐1α activate *Tert* and *Prdm10* remain uncertain. Finally, the potential for reciprocal regulation or synergistic interactions between *Tert* and *Prdm10* in DFA‐induced pluripotency maintenance requires further investigation.

## 4 Conclusion

**FIGURE 7:**
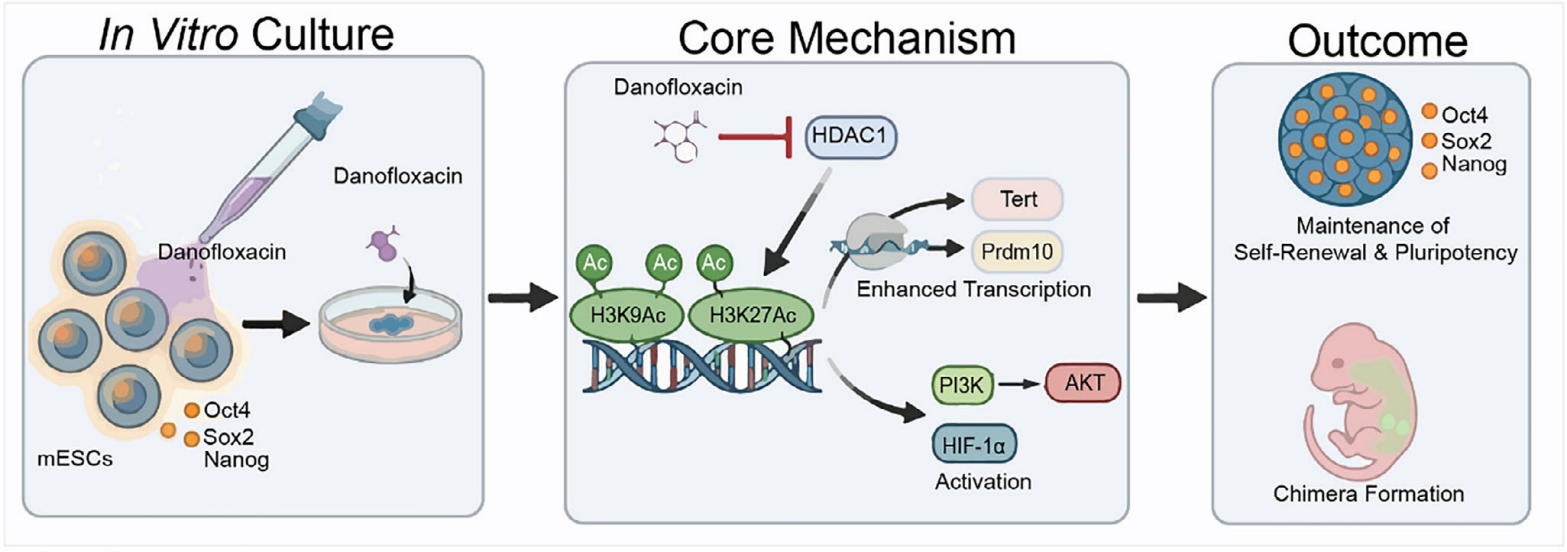
Proposed mechanism by which DFA maintains mESC stemness. DFA contributes to the maintenance of mESC self‐renewal and pluripotency and is accompanied by reduced *HDAC1* transcription. DFA treatment leads to increased H3K9ac and H3K27ac levels at the Tert and Prdm10 promoters, enhanced RNA polymerase II recruitment, and activation of the PI3K/AKT and HIF‐1α signaling pathways. These changes are associated with increased transcription of *Tert* and *Prdm10*.

In summary, this study identifies DFA as a self‐renewal‐promoting factor and elucidates its underlying mechanism, thereby providing an alternative strategy for stem cell culture. DFA treatment was associated with reduced *HDAC1* expression and increased acetylation of H3K9 and H3K27. These changes were accompanied by elevated expression of *Tert* and *Prdm10*, possibly in association with histone acetylation‐related recruitment of RNA polymerase II and changes in the PI3K/AKT and HIF‐1α signaling pathways (Figure 7). Together, these findings expand the current understanding of the stem cell regulatory network and offer a useful framework for developing strategies to maintain ESC pluripotency. Future comprehensive genome‐wide analyses, such as whole‐genome sequencing, will further strengthen the safety evaluation for translational applications.

## 5 Experimental Section

### 5.1 Cell Culture

46C mESCs, kindly provided by Qi‐Long Ying (University of Southern California, USA), were cultured in 0.1% gelatin‐coated dishes at 37°C in 5% carbon dioxide. The medium used for routine maintenance was DMEM (C3113‐0500, Vivacell, China) supplemented with 10% fetal bovine serum (FND500, ExCell Bio, Australia), 1×MEM nonessential amino acids (11140‐050, Gibco, USA), 2 mm GlutaMax (21051024, Gibco, USA) and 0.1 mm β‐mercaptoethanol (M3148, Sigma). Additionally, 1000 U/mL LIF (LIF1010, Millipore, USA) was added to sustain mESC stemness. The basal medium used to culture hiPSCs was N2B27 supplemented with 10% KnockOut Serum Replacement (KSR) (10828, Life Technologies). N2B27 consisted of the following components: Neurobasal medium (21103‐049, Life Technologies) and DMEM/F12 medium (11330‐032, Life Technologies) mixed at a 1:1 ratio; 1×non‐essential amino acids solution, 2 mm GlutaMAX, 0.1 mm β‐mercaptoethanol, 1×N2 (17502‐048, Life Technologies); and 1× B27 (17504‐044, Life Technologies). All cell lines used in this study were free from mycoplasma contamination for all experiments.

### 5.2 Plasmid Construction

The coding regions of the *Tert* and *Prdm10* genes were inserted into PiggyBac transposon vectors carrying a Flag tag. The primers for gene cloning are listed in Table S1. shRNA‐expressing plasmids were constructed according to the protocol for the pLKO.1‐TRC vector (Addgene plasmid #10878). Targeted specific shRNA sequences for Tert, Prdm10, HDAC1, and HIF‐1α are listed in Table S2.

### 5.3 AP activity Assay

Cells were fixed in 4% paraformaldehyde for 2 min at room temperature, followed by gentle washing with PBS three times. AP staining reagent (C3206, Beyotime Biotechnology, China) was prepared in the dark, and cells were incubated with the reagent at room temperature for 30 min. After incubation, cells were gently washed with PBS three times, and finally, the cells were visualized under a Leica DMI8 microscope.

### 5.4 Reverse Transcription–Quantitative PCR (RT‐qPCR)

Total RNA was extracted using the Cell/Tissue Total RNA Kit (19221ES50, YEASEN). 1 µg of total RNA was used to synthesize cDNA according to the instructions of HiScript III All‐in‐one RT SuperMix Perfect for qPCR (R333‐01, Vazyme). RT‐qPCR was performed using Hieff qPCR SYBR Green Master Mix (11201ES03, YEASEN) to prepare the PCR reaction mixture, and experiments were conducted on a PikoReal real‐time PCR machine (Thermo Scientific, USA). The relative expression levels were calculated using the 2^‐ΔΔCt^ method, with β‐actin expression used as the internal reference for normalization. The primers used in the experiment are listed in Table S3.

### 5.5 Western Blot

Cells were lysed using ice‐cold RIPA lysis buffer (P0013B, Beyotime Biotechnology, China) containing a protease inhibitor cocktail. The proteins were separated by electrophoresis on 10% SDS‐PAGE gels and subsequently transferred onto PVDF membranes. For antibody detection, specific primary antibodies were incubated overnight at 4°C, followed by incubation with horseradish peroxidase‐conjugated secondary antibodies at 25°C for 1 h. The primary antibodies used included Flag (F9291, Sigma,1:1000), β‐tubulin (250007, ZEN BIO,1:1000), Oct4 (11263‐1‐AP, Proteintech,1:1000), Klf4 (381633, ZEN BIO,1:1000), Sox2 (66411‐1‐Ig, Proteintech,1:1000), Nanog (14295‐1‐AP, Proteintech,1:1000), HDAC1 (10197‐1‐AP, Proteintech,1:1000), H3K9ac (ab4441, Abcam,1:1000), H3K27ac (ab4729, Abcam,1:1000), AKT (10176‐2‐AP, Proteintech,1:1000) and Pho‐AKT (66444‐1‐Ig, Proteintech,1:1000).

### 5.6 Immunofluorescence Staining

Immunostaining was performed according to the standard protocol. First, cells were fixed in 4% paraformaldehyde for 20 min, gently washed three times with PBS, and then incubated in blocking buffer (PBS containing 5% BSA and 0.2% Triton X‐100) at 37°C for 1 h. Subsequently, the cells were incubated overnight at 4°C with primary antibodies against Oct4 (11263‐1‐AP, Proteintech, 1:100), Sox2 (66411‐1‐Ig, Proteintech, 1:100), Tuj1 (R&D Systems, MAB1195, 1:100), Gata4 (Cell Signaling Technology, 36966S, 1:100), Myosin (Abcam, ab50967, 1:100), H3K9ac (ab4441, Abcam,1:200) and H3K27ac (ab4729, Abcam,1:200). After being gently washed three times with PBS, the cells were incubated with secondary antibody. The nuclei were stained with Hoechst 33342 (H3570, Invitrogen; 1:10,000).

### 5.7 CUT&Tag

The CUT&Tag experiment was performed according to the protocol of the Hyperactive Universal CUT&Tag Assay Kit for Illumina (Vazyme). Fresh and viable cells, approximately 50,000, were first collected and centrifuged at 300 × *g* for 2 min. The supernatant was discarded, and the cells were washed with pre‐prepared Wash Buffer, followed by another centrifugation at 300 × *g* for 2 min. The cells were then resuspended in Wash Buffer, and 10 µL of pre‐activated ConA beads were added to each sample. The mixture was incubated at room temperature for 10 min to form a cell‐bead complex. After discarding the supernatant, the cells were resuspended in 50 µL antibody buffer and incubated with 1 µg of primary antibodies (Anti‐H3K9ac and Anti‐H3K27ac). The mixture was incubated overnight at 4°C with gentle rotation. The next day, after centrifugation and removal of the supernatant, 50 µL of Dig‐wash buffer and 0.5‐1 µg of secondary antibody were added. The mixture was rotated at room temperature for 1 h, followed by three gentle washes with Dig‐wash buffer. Then, 2 µL of PA/G–Tnp and 98 µL of Dig‐300 buffer were added, and the mixture was incubated at room temperature for 1 h, followed by another three washes with Dig wash buffer. After discarding the supernatant, 40 µL of Dig‐300 buffer and 10 µL of 5 × TTBL were mixed, and the mixture was gently resuspended and incubated at 37°C for 1 h. DNA was extracted from the fragmented DNA using DNA Extract Beads and amplified through the appropriate cycles. The resulting library was purified using 2 × AMPure XP (Beckman Coulter). The library concentration was determined according to the Qubit dsDNA HS Kit (Invitrogen) protocol. The final CUT&Tag libraries were subjected to paired‐end sequencing on the NovaSeq 6000 platform (Illumina) according to the manufacturer's instructions.

### 5.8 Embryoid Bodies (EBs)

Spontaneous differentiation was induced by forming EBs through an 8‐day suspension culture on non‐adherent dishes. The EBs were then transferred to gelatin‐coated plates and cultured for another 5 days in mESC medium without LIF to promote further differentiation.

### 5.9 ULI‐NChIP‐seq

ULI‐NChIP‐seq was performed on 200–500 cells per sample with three biological replicates. Protein A beads (10 µL) were incubated with 1 µg of H3K4me3, H3K9me3, or H3K27me3 antibody at 4°C for at least 2 h. Cells were lysed in Nuclei Extraction Buffer and treated with MNase Master Mix at 25°C for 8 min. After stopping the reaction with EDTA and Nuclear Break Buffer, samples were brought to 180 µL with ChIP buffer, with 1/50 reserved as input. The remaining lysate was incubated with antibody‐coupled beads at 4°C for at least 4 h, then washed twice each with Low Salt and High Salt Wash Buffers. DNA was eluted at 65°C, purified by phenol‐chloroform extraction and isopropanol precipitation, then processed using the KAPA Hyper Prep kit. Libraries were sequenced on Illumina NovaSeq with 150‐bp paired‐end reads.

### 5.10 ChIP‐seq Data Analysis

ChIP‐seq data were analyzed following standard bioinformatics pipelines. Sequencing reads were trimmed using Trim Galore (v0.6.10) and aligned to the mouse reference genome (mm10) using Bowtie2 (v2.4.4). High‐confidence alignments (mapping_quality ≥ 20) were retained using Sambamba (v1.0.1), and PCR duplicates were removed using Picard (v3.1.0). Blacklist regions were filtered to eliminate artifacts. Normalized coverage tracks (bigWig) were generated using bamCoverage from deepTools (v3.5.4) with RPKM normalization. Background correction was performed by subtracting input signals using bigwigCompare. Peak calling was conducted using MACS2 (v2.2.7.1). Signal intensities were calculated using computeMatrix and visualized with plotHeatmap and plotProfile. Pearson correlation and PCA analyses assessed replicate consistency. ChIP‐seq signals were visualized using IGV (v2.16.0).

### 5.11 Quantitative Fluorescence In Situ Hybridization (Q‐FISH)

Q‐FISH experiments were performed using Cy3‐labeled PNA probes (0.7 µg/mL, Panagene) for telomere detection. Cells were first cultured in the presence of demecolcine (Sigma) for 4 h, followed by hypotonic swelling in 0.03 m sodium citrate at 37°C for 5 min. After two fixation changes, the cell suspension was dropped onto clean, cold microscopy slides and baked overnight at 65°C for 8–12 h. The slides were then soaked in PBS for 15 min, fixed in 4% formaldehyde for 2 min, and washed three times with PBS (5 min each). 15 µL of PNA probe was added to the slides and covered with a coverslip, then hybridized at 85°C for 10–15 min, followed by incubation at 18–25°C for 3 h. The coverslip was carefully removed with forceps, and the slides were washed in staining wash solution 1 (containing deionized formamide, BSA, and Tween 20) for 20 min, then in staining wash solution 2 (containing 5% dextrose) for 2 min. Finally, slides were counterstained with DAPI solution diluted in PBS at 18–25°C in the dark for 25 min. Images were acquired using an LSM 510 Meta confocal microscope equipped with a 63×/1.4 Oil DIC objective.

### 5.12 Dual‐Luciferase Reporter Assay

To assess the regulatory effect of DFA on HDAC1 promoter activity, the promoter sequence of mouse HDAC1 (spanning from −2000 to +1 bp) was cloned into the pGL6 reporter vector to generate the pGL6‐HDAC1 construct. 46C mESCs were co‐transfected with the pGL6‐HDAC1 plasmid and a Renilla luciferase internal control plasmid using Lipofectamine 3000. At 24 h post‐transfection, the culture medium was replaced with PDF medium, while cells maintained in PD medium served as the control group. Following an additional 24 h of incubation, luciferase activities were measured using the TransDetect Dual‐Luciferase Reporter Assay Kit (FR201, TransGen Biotech). The activity of the Firefly luciferase was normalized to that of the Renilla luciferase to determine the relative promoter activity. All assays were performed in at least three independent biological replicates to ensure statistical significance.

### 5.13 Chimera Generation

The microinjection technique was outlined in our previous report [10]. In brief, 8 to 10 single cells were injected into 3.5 dpc blastocysts obtained from ICR mice. The injected blastocysts were then transplanted into uterine horns of 2.5 d.p.c. pseudopregnant female mice. Chimeric offspring were subsequently identified based on coat color. For the generation of E12.5 chimeric embryos, two‐cell stage embryos were flushed from the oviducts of pregnant ICR mice using HCZB medium and transferred to G1‐plus medium covered with mineral oil, then cultured in vitro to the blastocyst stage. 8–10 GFP^+^ cells cultured in PDF condition were injected into each blastocyst‐stage embryo. After injection, the embryos were recovered in G1‐plus medium for 1–2 h, then 10–15 chimeric embryos were transferred into surrogate mice and dissected at the E12.5 developmental stage.

### 5.14 H&E Staining for E12.5 Mouse Embryos

E12.5 mouse embryos were dissected and immediately fixed in 4% PFA at 4°C overnight, washed three times with PBS, then dehydrated through a graded ethanol series (70%‐100%), 1 h per concentration. Samples were cleared in xylene three times (30 min each), infiltrated with xylene:paraffin (1:1) mixture and pure paraffin (58–60°C, 1 h per step), then embedded in paraffin blocks. Paraffin blocks were sectioned at 4–7 µm thickness, floated on a 40–45°C water bath, mounted onto slides, and baked at 65°C for 2 h. Sections were deparaffinized in xylene (twice, 10–15 min each), rehydrated through graded ethanol (100%–70%) to distilled water. Sections were stained with hematoxylin for 3 min, differentiated in 1% acid alcohol for 3–5 s, and blued in running water for 5–10 min. Sections were dehydrated in 95% ethanol for 1 min, then stained with eosin for 15 s to 1 min. Following staining, sections were dehydrated through graded ethanol (70%‐100%), cleared in xylene (twice, 5 min each), and mounted with neutral resin. Images were captured under a microscope. Staining showed nuclei in blue‐purple and cytoplasm in pink to red. Gentle handling is required as E12.5 embryonic tissue is small and soft.

### 5.15 Ethical Statement

All animal experiments involving the use of animals were approved by the Institutional Animal Care and Use Committee of Anhui University (No. IACUC(AHU)‐2024‐053).

### 5.16 Accession Number

Our RNA‐Seq dataset has been deposited in GEO under accession number GSE286230.

### 5.17 Statistical analysis

The number of biological replicates is indicated in each legend. All data are reported as the mean ± SD. The data were visualized with GraphPad Prism 8. Unpaired, two‐tailed Student's *t*‐tests were employed for comparisons between two groups. One‐way ANOVA or two‐way ANOVA was utilized for comparisons among multiple groups. A *p*‐value of less than 0.05 was considered statistically significant.

## Author Contributions

Y. Zhang and J. Ji designed the study. Y. Zhang and B. Dong collected data and performed the investigation. Z. Zhu performed bioinformatic analysis. X. Lv and P. Chen gave experimental technical support. Y. Zhang, W. Liu, M. Zhou, and S. D. Ye drafted the manuscript. All authors approved the final version of the manuscript for submission.

## Conflicts of Interest

The authors declare no conflicts of interest.

## Supporting information




**Supporting File**: advs77957‐sup‐0001‐SuppMat.docx.

## Data Availability

The data that support the findings of this study are available from the corresponding author upon reasonable request.

## References

[1] G. R. Martin , “Isolation of a Pluripotent Cell Line from Early Mouse Embryos Cultured in Medium Conditioned by Teratocarcinoma Stem Cells,” Proceedings of the National Academy of Sciences of the United States of America 78, no. 12 (1981): 7634–7638, 10.1073/pnas.78.12.7634. PMC349323 6950406

[2] M. J. Evans and M. H. Kaufman , “Establishment in Culture of Pluripotential Cells from Mouse Embryos,” Nature 292, no. 5819 (1981): 154–156, 10.1038/292154a0. 7242681

[3] A. M. Wobus , H. Holzhausen , P. Jakel , and J. Schoneich , “Characterization of a Pluripotent Stem Cell Line Derived from a Mouse Embryo,” Experimental Cell Research 152, no. 1 (1984): 212–219, 10.1016/0014-4827(84)90246-5. 6714319

[4] A. G. Smith , J. K. Heath , and D. D. Donaldson , “Inhibition of Pluripotential Embryonic Stem Cell Differentiation by Purified Polypeptides,” Nature 336, no. 6200 (1988): 688–690, 10.1038/336688a0. 3143917

[5] R. L. Williams , D. J. Hilton , and S. Pease , “Myeloid Leukaemia Inhibitory Factor Maintains the Developmental Potential of Embryonic Stem Cells,” Nature 336, no. 6200 (1988): 684–687, 10.1038/336684a0. 3143916

[6] G. Martello , P. Bertone , and A. Smith , “Identification of the Missing Pluripotency Mediator Downstream of Leukaemia Inhibitory Factor,” The EMBO Journal 32, no. 19 (2013): 2561–2574, 10.1038/emboj.2013.177. PMC3791366 23942233

[7] S. Ye , P. Li , C. Tong , and Q. L. Ying , “Embryonic Stem Cell Self‐Renewal Pathways Converge on the Transcription Factor Tfcp2l1,” The EMBO Journal 32, no. 19 (2013): 2548–2560, 10.1038/emboj.2013.175. PMC3791365 23942238

[8] C. I. Tai and Q. L. Ying , “Gbx2, a LIF/Stat3 Target, Promotes Reprogramming to and Retention of the Pluripotent Ground State,” Journal of Cell Science 126, no. 5 (2013): 1093–1098, 10.1242/jcs.118273. 23345404

[9] Q.‐L. Ying , J. Wray , J. Nichols , et al., “The Ground State of Embryonic Stem Cell Self‐Renewal,” Nature 453, no. 7194 (2008): 519–523, 10.1038/nature06968. PMC5328678 18497825

[10] Z. Zhu , Y. Zhang , X. Wang , X. Wang , and S. D. Ye , “Inhibition of Protein Kinase D by CID755673 Promotes Maintenance of the Pluripotency of Embryonic Stem Cells,” Development 147, no. 16 (2020): dev185264, 10.1242/dev.185264. 32747433

[11] X. Wang , X. Wang , X. Zhang , et al., “Inhibition of Ubiquitin‐Specific Protease 13‐Mediated Degradation of Raf1 Kinase by Spautin‐1 Has Opposing Effects in Naïve and Primed Pluripotent Stem Cells,” Journal of Biological Chemistry 297, no. 5 (2021): 101332, 10.1016/j.jbc.2021.101332. PMC8577099 34688658

[12] J. Wray , T. Kalkan , S. Gomez‐Lopez , et al., “Inhibition of Glycogen Synthase Kinase‐3 Alleviates Tcf3 Repression of the Pluripotency Network and Increases Embryonic Stem Cell Resistance to Differentiation,” Nature Cell Biology 13, no. 7 (2011): 838–845, 10.1038/ncb2267. PMC3160487 21685889

[13] G. Martello , T. Sugimoto , E. Diamanti , et al., “Esrrb Is a Pivotal Target of the Gsk3/Tcf3 Axis Regulating Embryonic Stem Cell Self‐renewal,” Cell Stem Cell 11, no. 4 (2012): 491–504, 10.1016/j.stem.2012.06.008. PMC3465555 23040478

[14] F. Yi , L. Pereira , and B. J. Merrill , “Tcf3 functions as a Steady‐State Limiter of Transcriptional Programs of Mouse Embryonic Stem Cell Self‐renewal,” Stem Cells 26, no. 8 (2008): 1951–1960, 10.1634/stemcells.2008-0229. PMC2743928 18483421

[15] J.‐C. Yeo , J. Jiang , Z.‐Y. Tan , et al., “Klf2 is an Essential Factor That Sustains Ground state Pluripotency,” Cell Stem Cell 14, no. 6 (2014): 864–872, 10.1016/j.stem.2014.04.015. 24905170

[16] J. Ji , J. Cao , P. Chen , R. Huang , and S. D. Ye , “Inhibition of Protein Kinase C Increases Prdm14 Level to Promote Self‐Renewal of Embryonic Stem Cells Through Reducing Suv39h‐Induced H3K9 Methylation,” Journal of Biological Chemistry 300, no. 3 (2024): 105714, 10.1016/j.jbc.2024.105714. PMC10909794 38309502

[17] T. Vazin and W. J. Freed , “Human Embryonic Stem Cells: Derivation, Culture, and Differentiation: A Review,” Restorative Neurology and Neuroscience 28, no. 4 (2010): 589–603, 10.3233/RNN-2010-0543. PMC2973558 20714081

[18] M. Buehr , S. Meek , K. Blair , et al., “Capture of Authentic Embryonic Stem Cells From Rat Blastocysts,” Cell 135, no. 7 (2008): 1287–1298, 10.1016/j.cell.2008.12.007. 19109897

[19] P. Li , C. Tong , R. Mehrian‐Shai , et al., “Germline Competent Embryonic Stem Cells Derived From Rat Blastocysts,” Cell 135, no. 7 (2008): 1299–1310, 10.1016/j.cell.2008.12.006. PMC2735113 19109898

[20] M. Kawamata and T. Ochiya , “Establishment of Embryonic Stem Cells From Rat Blastocysts,” Methods in Molecular Biology 597 (2010): 169–177, 10.1007/978-1-60327-389-3_12. 20013233

[21] X. Chen , Z. Guo , X. Tong , et al., “Derivation of Embryonic Stem Cells Across Avian Species,” Nature Biotechnology 44, no. 8 (2025): 1370–1382, 10.1038/s41587-025-02833-3. PMC13050621 41028831

[22] P. Chun‐on , A. M. Hinchie , H. C. Beale , et al., “TPP1 Promoter Mutations Cooperate with TERT Promoter Mutations to Lengthen Telomeres in Melanoma,” Science 378, no. 6620 (2022): 664–668, 10.1126/science.abq0607. PMC10590476 36356143

[23] T. J. Mitchell , S. Turajlic , A. Rowan , et al., “Timing the Landmark Events in the Evolution of Clear Cell Renal Cell Cancer: TRACERx Renal,” Cell 173, no. 3 (2018): 611–623, 10.1016/j.cell.2018.02.020. PMC5927631 29656891

[24] S. Bhatt and S. Chatterjee , “Fluoroquinolone Antibiotics: Occurrence, Mode of Action, Resistance, Environmental Detection, and Remediation—A Comprehensive Review,” Environmental Pollution 315 (2022): 120440, 10.1016/j.envpol.2022.120440. 36265724

[25] W. Lu , Y. Zhang , D. Liu , Z. Songyang , and M. Wan , “Telomeres—Structure, Function, and Regulation,” Experimental Cell Research 319, no. 2 (2013): 133–141, 10.1016/j.yexcr.2012.09.005. PMC4051234 23006819

[26] W. Palm and T. de Lange , “How Shelterin Protects Mammalian Telomeres,” Annual Review of Genetics 42, no. 1 (2008): 301–334, 10.1146/annurev.genet.41.110306.130350. 18680434

[27] T. M. Nakamura , G. B. Morin , K. B. Chapman , et al., “Telomerase Catalytic Subunit Homologs From Fission Yeast and Human,” Science 277, no. 5328 (1997): 955–959, 10.1126/science.277.5328.955. 9252327

[28] M. A. Blasco , “Telomeres and Human Disease: Ageing, Cancer and Beyond,” Nature Reviews Genetics 6, no. 8 (2005): 611–622, 10.1038/nrg1656. 16136653

[29] F. Wang , Y. Yin , X. Ye , et al., “Molecular Insights into the Heterogeneity of Telomere Reprogramming in Induced Pluripotent Stem Cells,” Cell Research 22, no. 4 (2012): 757–768, 10.1038/cr.2011.201. PMC3317559 22184006

[30] F. Pucci , L. Gardano , and L. Harrington , “Short Telomeres in ESCs Lead to Unstable Differentiation,” Cell Stem Cell 12, no. 4 (2013): 479–486, 10.1016/j.stem.2013.01.018. PMC3629568 23561444

[31] M. Criqui , A. Qamra , T. W. Chu , et al., “Telomere Dysfunction Cooperates with Epigenetic Alterations to Impair Murine Embryonic Stem Cell Fate Commitment,” Elife 9 (2020), e47333, 10.7554/eLife.47333. PMC7192583 32297856

[32] C. C. Liu , D. L. Ma , T. D. Yan , et al., “Distinct Responses of Stem Cells to Telomere Uncapping‐A Potential Strategy to Improve the Safety of Cell Therapy,” Stem Cells 34, no. 10 (2016): 2471–2484, 10.1002/stem.2431. 27299710

[33] S. Kyo , “Sp1 Cooperates with c‐Myc to Activate Transcription of the Human Telomerase Reverse Transcriptase Gene (hTERT),” Nucleic Acids Research 28, no. 3 (2000): 669–677, 10.1093/nar/28.3.669. PMC102554 10637317

[34] M. Vervoort , D. Meulemeester , J. Behague , and P. Kerner , “Evolution of Prdm Genes in Animals: Insights from Comparative Genomics,” Molecular Biology and Evolution 33, no. 3 (2016): 679–696, 10.1093/molbev/msv260. PMC4760075 26560352

[35] J. A. Park and K. C. Kim , “Expression Patterns of PRDM10 During Mouse Embryonic Development,” BMB Reports 43, no. 1 (2010): 29–33, 10.5483/bmbrep.2010.43.1.029. 20132732

[36] M. E. Dickinson , A. M. Flenniken , X. Ji , et al., “High‐throughput Discovery of Novel Developmental Phenotypes,” Nature 537, no. 7621 (2016): 508–514, 10.1038/nature19356. PMC5295821 27626380

[37] B. Y. Han , M. K. Y. Seah , I. R. Brooks , et al., “Global Translation During Early Development Depends on the Essential Transcription Factor PRDM10,” Nature Communications 11, no. 1 (2020): 3603, 10.1038/s41467-020-17304-3. PMC7368010 32681107

[38] N. Chen , T. Hu , Y. Gui , J. Gao , Z. Li , and S. Huang , “Transcriptional Regulation of Bcl‐2 Gene by the PR/SET Domain family Member PRDM10,” PeerJ 7 (2019): 6941, 10.7717/peerj.6941. PMC6525587 31143550

[39] P. Ma and R. M. Schultz , “Histone Deacetylase 1 (HDAC1) Regulates Histone Acetylation, Development, and Gene Expression in Preimplantation Mouse Embryos,” Developmental Biology 319, no. 1 (2008): 110–120, 10.1016/j.ydbio.2008.04.011. PMC2475681 18501342

[40] D. Willis‐Martinez , H. W. Richards , N. A. Timchenko , and E. E. Medrano , “Role of HDAC1 in Senescence, Aging, and Cancer,” Experimental Gerontology 45, no. 4 (2010): 279–285, 10.1016/j.exger.2009.10.001. PMC2838962 19818845

[41] B. L. Kidder and S. Palmer , “HDAC1 Regulates Pluripotency and Lineage Specific Transcriptional Networks in Embryonic and Trophoblast Stem Cells,” Nucleic Acids Research 40, no. 7 (2012): 2925–2939, 10.1093/nar/gkr1151. PMC3326306 22156375

[42] O. M. Dovey , C. T. Foster , and S. M. Cowley , “Histone Deacetylase 1 (HDAC1), but Not HDAC2, Controls Embryonic Stem Cell Differentiation,” Proceedings of the National Academy of Sciences 107, no. 18 (2010): 8242–8247, 10.1073/pnas.1000478107. PMC2889513 20404188

[43] S. Jamaladdin , R. D. W. Kelly , L. O'Regan , et al., “Histone Deacetylase (HDAC) 1 and 2 Are Essential for Accurate Cell Division and the Pluripotency of Embryonic Stem Cells,” Proceedings of the National Academy of Sciences 111, no. 27 (2014): 9840–9845, 10.1073/pnas.1321330111. PMC4103379 24958871

[44] J. Liang , M. Wan , Y. Zhang , et al., “Nanog and Oct4 Associate With Unique Transcriptional Repression Complexes in Embryonic Stem Cells,” Nature Cell Biology 10, no. 6 (2008): 731–739, 10.1038/ncb1736. 18454139

[45] J. Krejčí , R. Uhlirová , G. Galiová , S. Kozubek , J. Smigová , and E. Bártová , “Genome‐Wide Reduction in H3K9 Acetylation During Human Embryonic Stem Cell Differentiation,” Journal of Cellular Physiology 219, no. 3 (2009): 677–687, 10.1002/jcp.21714. 19202556

[46] J. H. Lee , S. R. Hart , and D. G. Skalnik , “Histone Deacetylase Activity Is Required for Embryonic Stem Cell Differentiation,” Genesis 38, no. 1 (2004): 32–38, 10.1002/gene.10250. 14755802

[47] C. B. Ware , L. Wang , B. H. Mecham , et al., “Histone Deacetylase Inhibition Elicits an Evolutionarily Conserved Self‐renewal Program in Embryonic Stem Cells,” Cell Stem Cell 4, no. 4 (2009): 359–369, 10.1016/j.stem.2009.03.001. PMC2719860 19341625

[48] N. V. Balmer , M. K. Weng , B. Zimmer , et al., “Epigenetic Changes and Disturbed Neural Development in a human Embryonic Stem Cell‐based Model Relating to the Fetal Valproate Syndrome,” Human Molecular Genetics 21, no. 18 (2012): 4104–4114, 10.1093/hmg/dds239. 22723015

[49] Y. Qiao , R. Wang , X. Yang , K. Tang , and N. Jing , “Dual Roles of Histone H3 Lysine 9 Acetylation in Human Embryonic Stem Cell Pluripotency and Neural Differentiation,” Journal of Biological Chemistry 290, no. 4 (2015): 2508–2520, 10.1074/jbc.M114.603761. PMC4303699 25519907

[50] D. Huangfu , K. Osafune , R. Maehr , et al., “Induction of Pluripotent Stem Cells From Primary Human Fibroblasts with Only Oct4 and Sox2,” Nature Biotechnology 26, no. 11 (2008): 1269–1275, 10.1038/nbt.1502. 18849973

[51] L. Zhu , Z. Liu , Y. Ren , et al., “Neuroprotective Effects of Salidroside on Ageing Hippocampal Neurons and Naturally Ageing Mice via the PI3K/Akt/TERT Pathway,” Phytotherapy Research 35, no. 10: 5767–5780, doi: 10.1002/ptr.7235. 10.1002/ptr.7235 34374127

[52] H. W. Tan , A. Y. Sim , S. L. Huang , Y. Leng , and Y. C. Long , “HC Toxin (a HDAC Inhibitor) Enhances IRS1–Akt Signalling and Metabolism in Mouse Myotubes,” Journal of Molecular Endocrinology 55, no. 3 (2015): 197–207, 10.1530/JME-15-0140. 26373795

[53] T. C. Zhao , J. Du , S. Zhuang , P. Liu , and L. X. Zhang , “HDAC Inhibition Elicits Myocardial Protective Effect Through Modulation of MKK3/Akt‐1,” PLoS ONE 8, no. 6 (2013): 65474, 10.1371/journal.pone.0065474. PMC3677871 23762381

[54] L. Xu , Q. Xing , T. Huang , et al., “HDAC1 Silence Promotes Neuroprotective Effects of Human Umbilical Cord‐Derived Mesenchymal Stem Cells in a Mouse Model of Traumatic Brain Injury via PI3K/AKT Pathway,” Frontiers in Cellular Neuroscience 12 (2018): 498, 10.3389/fncel.2018.00498. PMC6328439 30662396

[55] H. Niwa , K. Ogawa , D. Shimosato , and K. Adachi , “A Parallel Circuit of LIF Signalling Pathways Maintains Pluripotency of Mouse ES Cells,” Nature 460, no. 7251 (2009): 118–122, 10.1038/nature08113. 19571885

[56] A. M. Singh , D. Reynolds , T. Cliff , et al., “Signaling Network Crosstalk in Human Pluripotent Cells: A Smad2/3‐regulated Switch That Controls the Balance Between Self‐renewal and Differentiation,” Cell Stem Cell 10, no. 3 (2012): 312–326, 10.1016/j.stem.2012.01.014. PMC3294294 22385658

[57] H. Lu , Y. Lyu , L. Tran , et al., “HIF‐1 Recruits NANOG as a Coactivator for TERT Gene Transcription in Hypoxic Breast Cancer Stem Cells,” Cell Reports 36, no. 13 (2021): 109757, 10.1016/j.celrep.2021.109757. 34592152

[58] H. J. Lee , Y. H. Jung , G. E. Choi , J. S. Kim , C. W. Chae , and H. J. Han , “Role of HIF1 α Regulatory Factor in Stem Cells,” International Journal of Stem Cells 12, no. 1 (2019): 8–20, 10.15283/ijsc18109. PMC6457711 30836734

[59] K.‐M. Ng , Y.‐K. Lee , Y.‐C. Chan , et al., “Exogenous Expression of HIF‐1α Promotes Cardiac Differentiation of Embryonic Stem Cells,” Journal of Molecular and Cellular Cardiology 48, no. 6 (2010): 1129–1137, 10.1016/j.yjmcc.2010.01.015. 20116384

[60] W. Zhou , M. Choi , D. Margineantu , et al., “HIF1α Induced Switch from Bivalent to Exclusively Glycolytic Metabolism During ESC‐to‐EpiSC/hESC Transition,” The EMBO Journal 31, no. 9 (2012): 2103–2116, 10.1038/emboj.2012.71. PMC3343469 22446391

[61] H. J. Lee and K. W. Kim , “Suppression of HIF‐1α by Valproic Acid Sustains Self‐Renewal of Mouse Embryonic Stem Cells Under Hypoxia In Vitro,” Biomolecules and Therapeutics 20, no. 3 (2012): 280–285, 10.4062/biomolther.2012.20.3.280. PMC3794524 24130924

