# Supplementary material for: Chemical Screening Identifies Danofloxacin as a Self‐Renewal Agonist of Embryonic Stem Cells through Alleviation of HDAC1‐Mediated Deacetylation of Tert and Prdm10
Source: Adv Sci (Weinh). 2026 Sep 27:e00624. Online ahead of print. doi: 10.1002/advs.202500624 (PMC13616382; doi:10.1002/advs.202500624)
Supplement: Supplementary file 1 [file ADVS-9999-e00624-s001.docx]

Supporting Information

Chemical screening identifies Danofloxacin as a self-renewal agonist of embryonic stem cells through alleviation of HDAC1-mediated deacetylation of *Tert* and *Prdm10*

Yan Zhang, Junxiang Ji, Baoxing Dong, Zhenhua Zhu, Xucheng Lv, Peng Chen, Wenqiang Liu, Min Zhou, Shou-Dong Ye

**This file includes:**

**Supplementary Figures and Figure legends**

**Supplementary Tables**

**Supplementary Figures and Figure legends**

**Figure S1.** Identification and characterization of DFA-mediated mESC self-renewal. A) Flow cytometry analysis of *Rex1*-GFP reporter mESCs cultured for 7 days in 10% serum-containing medium without LIF, comparing untreated cells with those treated with 5 μM DFA. B) Quantification of AP-positive colonies from Figure 1A. C) AP-positive colonies from Figure 1B. D) Quantification of AP-positive colonies from Figure 1C. E) Quantification of fluorescence intensity (Green) in Figure 1D using ImageJ software (n=3). F) RT-qPCR analysis of *Gbx2*, *Klf4*, *Tfcp2l1*, *Klf2*, *Klf5*, and *Oct4* expression in 46C mESCs cultured in PD or PDF (n=3). G) Chimera efficiency corresponding to the data shown in Figure 1M. The data are presented as mean ± SD. Statistical analyses were performed using one-way ANOVA (E) and Student’s *t*-test (F). **p* < 0.05, ***p* < 0.01, ****p* < 0.001, *****p* < 0.0001.

**Figure S2.** DFA promotes short-term self-renewal in human iPSCs. A) AP staining of human iPSCs cultured for 5 days in KSR-containing medium with a gradient of DFA (1, 1.5, 2.5, and 3 μM). Scale bar: 100 μm. B) Quantification of AP-positive colonies from (A). C) Immunofluorescence staining of Sox2 in human iPSCs cultured under a gradient of DFA (1, 1.5, 2, and 2.5 μM). Scale bar: 100 μm. D) Quantification of fluorescence intensity (Green) in (C) using ImageJ (n=3). E) AP staining of human iPSCs cultured for 7 days in KSR-containing medium with 2.5 μM DFA. Scale bar: 100 μm. F) Quantification of AP-positive colonies from (E). G) RT-qPCR analysis of the expression of OCT4 and NANOG in human iPSCs treated with or without DFA (n=3). H) RT-qPCR analysis of the expression of *TERT* and *PRDM10* in human iPSCs treated with PD or PDF (n=3). The data are presented as mean ± SD. Statistical analyses were performed using Student’s *t*-test (G) and one-way ANOVA (D,H). **p* < 0.05, ****p* < 0.001, *****p* < 0.0001.

**Figure S3.** Functional validation of Tert and Prdm10 in mESC maintenance. A) Densitometric analysis of the relative protein levels of Oct4, Klf4, and Nanog (corresponding to Figure 3C) using ImageJ. Protein levels were normalized to β-tubulin (n=3). B) Densitometric analysis of the relative protein levels of Oct4, Klf4, and Nanog (corresponding to Figure 3E) using ImageJ. Protein levels were normalized to β-tubulin (n=3). C)AP staining of mESCs overexpressing *FLAG*, *F-Tert*, or *F-Prdm10*, cultured for 7 days in 10% serum-containing medium without LIF. Scale bar: 100 μm. D) Quantification of AP-positive colonies from Figure S3C. E) Quantification of AP-positive colonies from Figures 3I and 3J. F) RT-qPCR analysis of telomere repeat length in 46C mESCs overexpressing *F-Tert* or *FLAG* (n=3). G) RT-qPCR analysis of telomere repeat length in 46C mESCs treated with PD or PDF (n=3). The data are presented as mean ± SD. Statistical analyses were performed using two-way ANOVA (A,B) and Student’s *t*-tests (F,G). **p* < 0.05, ***p* < 0.01, *****p* < 0.0001.

**Figure S4.** Target prediction and HDAC1 inhibitory mechanism of DFA. A) Chemical structure of DFA. B) Classification of DFA target proteins predicted using the SwissTargetPrediction database. C) Densitometric analysis of HDAC1 protein levels (from Figure 4A) using ImageJ. Levels were normalized to β-tubulin (n=3). D) Quantification of AP-positive colonies from Figure 4B. E,F) Molecular docking model illustrating predicted binding sites and interactions between DFA and HDAC1. G) Relative HDAC1 activity in mESCs treated with TSA or DFA for 0.5 hours (n=3). H) Densitometric analysis of HDAC1 protein levels (from Figure 4C) using ImageJ. Levels were normalized to β-tubulin (n=3). I) Densitometric analysis of H3K9ac and H3K27ac protein levels (from Figure 4E) using ImageJ. Levels were normalized to β-tubulin (n=3). J) Western blot analysis of H3K9ac and H3K27ac levels in mESCs treated with different durations of TSA. β-tubulin served as the loading control. K) Densitometric analysis of H3K9ac and H3K27ac protein levels from Figure S4J using ImageJ. Levels were normalized to β-tubulin (n=3). L) Genome browser tracks showing enrichment of H3K9me3, H3K27me3, and H3K4me3 at the *HDAC1* locus in PD and PDF culture conditions. Highlighted regions indicate differential enrichment between conditions. M,N) Luciferase reporter assay of *HDAC1* promoter activity in cells treated with PD or PDF (n=3). The data are presented as mean ± SD. Statistical analyses were performed using one-way ANOVA (C,G,K), Student’s *t*-test (H,N) and two-way ANOVA (I). **p* < 0.05, ***p* < 0.01, ****p* < 0.001, *****p* < 0.0001.

**Figure S5.** Dynamics of HDAC1 expression. A) Densitometric analysis of HDAC1 protein levels from Figure 5A using ImageJ. Values were normalized to β-tubulin (n=3). B) RT-qPCR analysis of *HDAC1* expression in scramble *control* and *HDAC1*-knockdown (*HDAC1* sh#1, *HDAC1* sh#2) 46C mESCs. (n=3). The data are presented as mean ± SD. Statistical analyses were performed using one-way ANOVA (A,B). ***p* < 0.01, ****p* < 0.001, *****p* < 0.0001.

**Figure S6.** Effects of DFA and TSA on PI3K/AKT signaling pathway. A) Densitometric analysis of AKT and phosphorylated AKT (Pho-AKT) protein levels from Figure 6E using ImageJ. Values were normalized to β-tubulin (n=3). B) Western blot analysis of the levels of AKT and Pho-AKT in mESCs treated with or without TSA. The data are presented as mean ± SD. Statistical analyses were performed using one-way ANOVA. **p* < 0.05, ****p* < 0.001, *****p* < 0.0001.

**Supplementary Tables**

Table S1. Primer sequences for gene overexpression experiments

| Symbol (Mouse) | Forward sequence (5'-3’) | Reverse sequence (5'-3’) |
| --- | --- | --- |
| *Tert* | ATGCGGCCCCTGTTCCAACAGCTGC | TTAGTCCAAAATGGTCTGAAAGTCT |
| *Prdm10* | ATGGATCCCAAAGATGAAAGCGCTC | GGCTGGGGCCTGTGGAGTCCAGGGA |

Table S2. List of knockdown sequences used for gene knockdown

| Symbol (Mouse) | Forward sequence (5'-3’) |
| --- | --- |
| *HDAC1* sh#1 | GCCACCATCATTGATCTCGAT |
| *HDAC1* sh#2 | CCATGATATACCCATCCTCAT |
| *Tert* sh#1 | CGCCGCTTCTTTAAGAACTTA |
| *Tert* sh#2 | GCTCATTCTGTCATCTACAAA |
| *Prdm10* sh#1 | ATGAGAAACTGGACGTGTTTA |
| *Prdm10* sh#2 | AGTTGACCCTGCCCATCATAA |
| *HIF-1α* sh#1 | ATTTCTTTGATTCAAAGTGGC |
| *HIF-1α* sh#2 | AATATTCATAAATTGAGCGGC |

Table S3. List of primers used for RT-qPCR analysis

| Symbol  (Mouse) | Forward sequence (5'-3’) | Reverse sequence (5'-3’) |
| --- | --- | --- |
| *β-actin* | TATCGCTGCGCTGGTCG | CCCACGATGGAGGGGAATAC |
| *Gbx2* | AGTGGAAACGTGTCAAGGCA | AGCGAACCTGCTAACGTGAA |
| *Klf4* | CTCAGACTCCACTGCACACC | CTCCACAGCACGAGCTTTCT |
| *Tfcp2l1* | GTGGGGACTACTCGGAGCAT | AAGCTGTTGGGAGAGCCATT |
| *Klf2* | GCGTACACACACAGGTGAGA | GCACAAGTGGCACTGAAAGG |
| *Klf5* | CCGTCCTATGCCGCTACAAT | CTTCTCGCCCGTATGAGTCC |
| *Oct4* | GAAGCAGAAGAGGATCACCTTG | TTCTTAAGGCTGAGCTGCAAG |
| *Sox15* | TCCCTCAGAGTGATCCCAGG | AAGGTGGGTTACTGGCATGG |
| *Tert* | AACACCTTGAGAGAGTGCGG | GTCTTGAGACGCTGGGTGAA |
| *Trim43b* | TCCATGAAGCTACGGGAGGA | AGCTCCCGATACACCTCTGT |
| *Prdm10* | TCAGCCCCTCCCATATCCAA | CTGGGGGCAGTGTCATCATT |
| *Nanog* | GTCTGATTCAGGGCTCAGCA | AAGGCTTCCAGATGCGTTCA |
| *Rex1* | TCACTGTGCTGCCTCCAAGT | GGGCACTGATCCGCAAAC |
| *Sox2* | GGAGAACCCCAAGATGCACA | GCTTCTCGGTCTCGGACAAA |
| *Gata4* | TCTCACTATGGGCACAGCAG | GCGATGTCTGAGTGACAGGA |
| *Gata6* | TCCTCCCCTGCCGAAGTC | AGGGCCAGAGCACACCAA |
| *Cdx2* | ACCGGAATTGTTTGCTGCTGT | TCCCGACTTCCCTTCACCAT |
| *Tbx3* | CTTTCGGGAGCCTGTTTCCT | ACTGGGACGGGGATGGAATA |
| *Esrrb* | GAACAGCCCCTACCTGAACC | ATGAGGAACACAAGCTCCCG |
| *Otx2* | TGAGGCCTGCCAAGAAGAAG | GACAAGGGGTCAGACAGTGG |
| *HDAC1* | TGTTCCAGCCTAGTGCAGTG | CACACTTGGCGTGTCCTTTG |
| *HIF-1α* | GGCTCCCTTTTTCAAGCAGC | CGTAACTGGTCAGCTGTGGT |
